# In Vitro Inhibition of *Phytophthora nicotianae* by Berberine and Associated Physiological and Transcriptomic Responses

**DOI:** 10.3390/jof12100745

**Published:** 2026-10-04

**Authors:** Zaiqian Xu, Zhiyong Tan, Qi Hu, Yuzhu Li

**Affiliations:** 1 College of A&F Engineering and Planning, Tongren University, Tongren 554300, China; 18768139120@163.com (Z.X.); 15736342621@163.com (Q.H.); 2 Institute of Resources and Environment, Tongren University, Tongren 554300, China; 3 College of Environment and Ecology, Hunan Agricultural University, Changsha 410128, China; 15254051332@163.com

**Keywords:** berberine, *Phytophthora nicotianae*, anti-oomycete activity, membrane integrity, energy metabolism, transcriptomics

## Abstract

*Phytophthora nicotianae* is a destructive plant-pathogenic oomycete. This study evaluated the in vitro inhibitory activity of berberine against strain ACCC 39210 and examined associated physiological and transcriptomic responses. Berberine inhibited mycelial growth, with an estimated EC_50_ of 42.1 μg/mL (95% CI: 7.66–76.55 μg/mL). Exposure to 42 and 200 μg/mL increased malondialdehyde (MDA) content and superoxide dismutase activity, while decreasing peroxidase activity, ATPase activity, and ATP content. Catalase activity only decreased significantly at 200 μg/mL. Conductivity measurements, propidium iodide staining, and microscopy provided evidence of cellular-envelope injury. Exploratory RNA-sequencing analysis using unadjusted *p* < 0.05 and |log2(fold change)| > 1 identified 1255 and 1417 candidate differentially expressed genes at 42 and 200 μg/mL, respectively. Functional annotations suggested changes in metabolism, transport, and stress responses. Quantitative reverse-transcription PCR showed concordant expression directions for eight selected genes, using the same biological samples and a single reference gene; this comparison did not provide independent biological validation. These findings associate berberine-mediated growth inhibition with cellular-envelope injury, altered antioxidant enzyme activities, reduced ATP availability, and transcriptional responses in one isolate. Causal mechanisms, efficacy in planta, crop safety, and sensitivity across isolates require further investigation.

## 1. Introduction

*Phytophthora nicotianae* is a polyphagous oomycete that causes destructive diseases in more than 255 genera across 90 plant families, including economically important fruit, vegetable, forestry, and ornamental crops [1]. In tobacco, the pathogen causes black shank and can markedly reduce plant health and productivity [2]. Disease management still relies heavily on synthetic fungicides, but repeated use can select resistant pathogen populations and contribute to residues and environmental contamination [3,4]. Plant-derived compounds with anti-oomycete activity therefore warrant investigation.

Berberine (C_20_H_18_NO_4_; molecular weight, 336.37) is a naturally occurring isoquinoline alkaloid found in medicinal plants such as *Coptis chinensis*, *Phellodendron chinense*, and *Berberis* species. Its broad pharmacological and antimicrobial properties have been widely reviewed [5,6,7], and direct evidence of activity against plant-pathogenic fungi is available. For example, berberine inhibited *Penicillium italicum* growth and spore germination and suppressed blue mold development in citrus fruit [8]. These findings motivate evaluation against oomycetes but do not establish efficacy or crop safety in tobacco.

Mechanistic studies suggest that berberine can reduce hyphal lipid content, disrupt plasma-membrane integrity, and promote leakage of intracellular constituents [9]. Berberine inhibits the development of *Monilinia fructicola* [10]. In a separate study, berberine reduced the activity of recombinant cutinase from *Colletotrichum capsici*, and molecular simulations suggested possible interactions underlying this effect [11]. Nevertheless, its activity against *P. nicotianae* and the integrated physiological and transcriptional responses of this oomycete have not been characterized in detail.

This study therefore evaluated the concentration-dependent in vitro activity of berberine against *P. nicotianae* and examined changes in mycelial biomass, membrane integrity, cell-wall-associated staining, malondialdehyde (MDA) content, antioxidant enzyme activities, ATPase activity, ATP content, and global gene expression. Selected candidate genes were further assessed by qRT-PCR. By integrating these measurements, the study aimed to characterize physiological stress responses and generate hypotheses about the processes associated with growth inhibition, providing a foundation for future mechanistic studies and in planta efficacy evaluation. MDA is a product of lipid peroxidation and is used as an indirect marker of oxidative lipid injury; changes in MDA content therefore provide context for membrane-related alterations but do not by themselves establish their cause.

## 2. Materials and Methods

### 2.1. Pathogen Strain and Culture Media

*Phytophthora nicotianae* strain ACCC 39210 was obtained from the Agricultural Culture Collection of China (ACCC), Beijing, China, and used throughout this study. The strain was maintained on oatmeal agar (OA; 30 g oatmeal and 15 g agar (Kermel Chemical Technology Co., Ltd., Tianjin, China) per liter of distilled water) and subcultured before each experiment. Oatmeal broth (OB) contained 30 g oatmeal per liter of distilled water.

### 2.2. Experimental Procedures

#### 2.2.1. In Vitro Mycelial Growth Inhibition

The inhibitory activity of berberine (97% purity; Shanghai Yien Chemical Technology Co., Ltd., Shanghai, China) was evaluated using a radial growth assay. Uniform 5 mm plugs were excised from the margins of actively growing colonies and placed on OA plates containing 0, 6.25, 12.5, 25, 50, 100, or 200 μg/mL berberine. Berberine-free OA served as the untreated control (CK). Plates were sealed with Parafilm (Amcor, Neenah, WI, USA), incubated upside down at 28 °C for 4 d, and assessed by measuring two perpendicular colony diameters. Each treatment comprised three biological replicates.

Mycelial growth inhibition (%) = [(Dc − Dt)/Dc ] × 100, where Dc and Dt are the mean final colony diameters of the untreated control and berberine treatment, respectively [12].

The concentration–response relationship was fitted using a four-parameter log−logistic model (LL.4) implemented in the drc 3.0.1 package in R 4.4.3. All four model parameters were estimated from the mean mycelial growth inhibition values at six nonzero berberine concentrations. The absolute EC_50_, defined as the concentration corresponding to 50% mycelial growth inhibition relative to the untreated control, was estimated using the ED function with type = “absolute”. Its standard error and 95% confidence interval were calculated using the delta method. Descriptive goodness-of-fit was expressed as pseudo-R^2^, calculated as 1 − RSS/TSS, where RSS and TSS denote the residual and total sums of squares, respectively, on the inhibition-response scale [13].

Based on the four-parameter logistic model, the estimated EC_50_ was 42.1 μg/mL; this value was rounded to 42 μg/mL for use as the nominal EC_50_ treatment concentration in subsequent experiments.

#### 2.2.2. Mycelial Dry Weight

Berberine was dissolved in OB pre-warmed to 80 °C to obtain final concentrations of 6.25, 12.5, 25, 50, 100, and 200 μg/mL. Six standardized mycelial plugs were inoculated into each flask; berberine-free OB served as CK. Cultures were incubated at 160 rpm for 7 d in an incubated orbital shaker (Shanghai Chubai Instrument Co., Ltd., Shanghai, China). Mycelia were then harvested, rinsed with phosphate-buffered saline (PBS, 0.01 mol/L, pH 7.2–7.4; Guangzhou Hewei Pharmaceutical Technology Co., Ltd., Guangzhou, China), and oven-dried at 60 °C to constant weight. Three biological replicates were used for each treatment [14].

#### 2.2.3. Malondialdehyde Content

Malondialdehyde (MDA) content was determined using a commercial assay kit (BC0025; Beijing Solarbio Science & Technology Co., Ltd., Beijing, China). After incubation without berberine (CK) or with 42 or 200 μg/mL berberine at 28 °C for 24 h, 0.9 g of fresh mycelia from each sample was collected and mixed with 3 mL of the extraction solution supplied with the kit. The samples were homogenized on ice, and the homogenates were subsequently processed according to the manufacturer’s instructions. Absorbance was measured at 532 and 600 nm using a microplate reader (Thermo Fisher Scientific (Shanghai) Instruments Co., Ltd., Shanghai, China). MDA content was calculated according to the kit instructions [15].

#### 2.2.4. Antioxidant Enzyme Activities

The activities of superoxide dismutase (SOD), peroxidase (POD), and catalase (CAT) were determined using commercial assay kits (SOD, BC0175; POD, BC0095; CAT, BC0205; Beijing Solarbio Science & Technology Co., Ltd., Beijing, China) with reference to a previously reported method [16]. Mycelia were incubated without berberine (CK) or with 42 or 200 μg/mL berberine at 28 °C for 24 h. After treatment, enzyme extracts were prepared and the activities of SOD, POD, and CAT were determined according to the manufacturer’s instructions. Three independent biological replicates were analyzed for each treatment.

#### 2.2.5. ATPase Activity and ATP Content

After 24 h of incubation without berberine (CK) or with 42 or 200 μg/mL berberine, mycelial extracts were prepared as specified by the manufacturer. ATPase activity (A070-1-1) and ATP content (A095-1-1) were determined separately using colorimetric kits (Nanjing Jiancheng Bioengineering Institute, Nanjing, China) according to the manufacturer’s instructions, with values normalized to protein content [17]. Absorbance was measured at 636 nm using a microplate reader (Thermo Fisher Scientific (Shanghai) Instruments Co., Ltd., Shanghai, China). Three independent biological replicates were analyzed per treatment.

#### 2.2.6. Relative Electrical Conductivity

Relative electrical conductivity was measured using a conductivity meter (INESA Scientific Instrument Co., Ltd., Shanghai, China) according to a previously reported method [18]. Briefly, 0.1 g of fresh mycelia was immersed directly in 30 mL distilled water supplemented with 0 (CK), 42, or 200 μg/mL berberine. Initial conductivity (L0) was recorded separately for each group, and conductivity at time t (L1) was measured after 0.5, 1.0, 1.5, 2.0, 2.5, and 3.0 h. After the final measurement, samples were boiled, cooled, and measured again to obtain total conductivity (L2). Relative electrical conductivity was calculated as [(L1 − L0)/(L2 − L0)] × 100. Each treatment was performed in triplicate.

#### 2.2.7. Cell-Wall-Associated Staining

Mycelia were incubated without berberine (CK) or with 42 or 200 μg/mL berberine for 24 h before staining. Mycelial samples were immersed in 200 μL of 10% aqueous tannic acid (AR grade, 250 g package; Guangfu, Tianjin, China) and incubated in the dark for 60 min. After three washes with sterile water, samples were stained with 5% aqueous crystal violet (AR grade, 25 g package; Guangfu, Tianjin, China) for 5 min, washed three times, stained with 5% aqueous Congo red (IND grade, 25 g package; Kermel, Tianjin, China) for 2 min, and washed again. Stained mycelia were mounted on glass slides and imaged with an inverted microscope (DM IL LED, Leica Microsystems CMS GmbH, Wetzlar, Germany) [19]. Three independent biological replicates were used per treatment.

#### 2.2.8. Propidium Iodide Staining

Mycelial membrane integrity was evaluated using propidium iodide (PI) staining, an approach previously applied to assess membrane damage in *P. nicotianae* [20]. Mycelia from the CK, 42 μg/mL, and 200 μg/mL berberine groups were washed three times with PBS and incubated with 200 μL of PI solution (40 μg/mL; Aladdin, Shanghai, China) at room temperature in the dark for 15 min. The PI-stained mycelia were mounted on glass slides and imaged using an inverted fluorescence microscope (Leica DM IL LED Fluo, Leica Microsystems, Wetzlar, Germany) equipped with an N2.1 filter cube (515–560 nm excitation bandpass filter, 580 nm dichroic mirror, and 590 nm long-pass emission filter). For each treatment, the bright-field and PI fluorescence images were acquired from the same field of view. Exposure and gain settings were kept consistent across treatments for each imaging mode.

#### 2.2.9. Scanning Electron Microscopy

After 24 h of incubation without berberine (CK) or with 42 or 200 μg/mL berberine, mycelia were fixed in 2.5% glutaraldehyde (EM grade; Beijing Solarbio Science & Technology Co., Ltd., Beijing, China) at 4 °C for 12 h. Samples were washed three times for 15 min with 0.1 mol/L phosphate buffer (pH 7.0) and dehydrated for 15 min at each step in 30%, 50%, 70%, 80%, 90%, and 95% ethanol (Sinopharm Chemical Reagent Co., Ltd., Shanghai, China), followed by two 20 min washes in 100% ethanol. After critical-point drying, samples were mounted on conductive carbon tape, sputter-coated with platinum for approximately 120 s, and examined with a field-emission scanning electron microscope (Regulus8100, Hitachi High-Tech Corporation, Tokyo, Japan) [16]. Three independent biological replicates were used per treatment.

#### 2.2.10. Transcriptome Sequencing and Bioinformatic Analysis

For transcriptome analysis, *P. nicotianae* was cultured for 24 h in 150 mL of oatmeal broth (OB) under one of three berberine conditions: no berberine (CK), 42 μg/mL (the nominal EC_50_ treatment), or 200 μg/mL. For each treatment, 0.3 g of mycelia was collected from each of three independent biological replicates (three separate flasks), yielding nine samples in total.

Total RNA was extracted using TRIzol reagent (Invitrogen, Thermo Fisher Scientific, Waltham, MA, USA). RNA concentration and purity were assessed using a NanoDrop One spectrophotometer (Thermo Fisher Scientific, Waltham, MA, USA), and RNA integrity was examined before library preparation. Poly(A)-tailed mRNA was enriched from total RNA using oligo(dT) magnetic beads and fragmented to approximately 300 bp. Strand-specific libraries were prepared using the NEBNext Ultra II RNA Library Prep Kit for Illumina (New England Biolabs, Ipswich, MA, USA). First-strand cDNA was synthesized using random primers, followed by second-strand cDNA synthesis with dUTP replacing dTTP. After adaptor ligation and PCR amplification, library fragments of approximately 400–500 bp were selected. Library fragment size and concentration were assessed before sequencing. Qualified libraries were subjected to paired-end 150 bp sequencing on an Illumina platform by Shanghai Personal Biotechnology Co., Ltd. (Shanghai, China).

After adaptor removal and quality filtering, clean reads were aligned to the *P. nicotianae* INRA-310 reference genome (assembly PP_INRA-310_V2; RefSeq accession GCF_000247585.1). Gene-level read counts were generated using HTSeq 0.9.1 [21], and FPKM values were calculated to represent normalized gene-expression levels. Differential expression analysis was performed using DESeq2 1.38.3 based on gene-level read counts [22]. The 42 and 200 μg/mL treatment groups were separately compared with the control group. An exploratory screening threshold of |log2(fold change)| > 1 and an unadjusted *p*-value < 0.05 was used to identify candidate differentially expressed genes (candidate DEGs). This screening threshold did not control the false discovery rate; the resulting candidate-gene lists were interpreted as exploratory.

GO and KEGG over-representation analyses of the candidate DEGs were performed using clusterProfiler 4.6.0 [23], with the corresponding genome-wide annotated genes used as the background set. Over-representation was assessed using a hypergeometric test, and terms or pathways with an unadjusted *p*-value < 0.05 were retained for exploratory interpretation. These results were not treated as enrichment findings supported by multiple-testing correction. For heatmap visualization, gene-expression values were transformed into Z-scores, and candidate DEGs and samples were subjected to two-way clustering. Genes with similar expression patterns across samples were grouped into expression clusters.

Principal-component analysis (PCA) was performed based on gene-expression values to visualize relationships among the nine RNA-seq samples. Three independent biological replicates were included for each treatment. Samples Y0_1–Y0_3 represented the untreated control, Y42_1–Y42_3 represented the 42 μg/mL berberine treatment, and Y200_1–Y200_3 represented the 200 μg/mL treatment. These groups were labeled CK, T1, and T2, respectively, in the PCA plot.

#### 2.2.11. Targeted qRT-PCR Analysis

Eight candidate genes were selected for a targeted qRT-PCR comparison with the RNA-seq expression patterns. The same biological samples were used for RNA-seq and qRT-PCR analyses. Total RNA was extracted as described in Section 2.2.10, and its quality was assessed by agarose-gel electrophoresis and using a NanoDrop 2000 spectrophotometer (Thermo Fisher Scientific, Wilmington, DE, USA). First-strand cDNA was synthesized from 1 μg of total RNA using an Advantage RT-for-PCR Kit (cat. no. 639505; Takara Bio Inc., Kusatsu, Japan) according to the manufacturer’s instructions, with incubation at 42 °C for 60 min followed by 94 °C for 5 min. qRT-PCR was performed on a LightCycler 480 II system (Roche, Basel, Switzerland) in 20 μL reactions containing 10.0 μL of 2× AceQ qPCR SYBR Green Master Mix (Q111-02; Vazyme, Nanjing, China), 0.4 μL of each primer (10 μmol/L), and 4.0 μL of diluted cDNA, with nuclease-free water added to a final volume of 20 μL. The amplification program consisted of 95 °C for 5 min, followed by 40 cycles of 95 °C for 15 s and 60 °C for 30 s. Amplification specificity was assessed by melting-curve analysis. *PPTG_16002*(actin) was used as the reference gene, and relative expression levels were calculated using the 2−ΔΔCt method with the untreated control as the calibrator [24]. Three independent biological replicates were analyzed per treatment, with three technical replicates for each sample–gene combination. Primer sequences are listed in Table 1.

### 2.3. Statistical Analysis

Data were analyzed using Excel 2022 and SPSS 27.0. The figure legends specify one-way ANOVA followed by Duncan’s multiple-range test for mycelial dry weight, MDA content, SOD, POD and CAT activities, ATPase activity, ATP content, and within-gene qRT-PCR comparisons. For relative electrical conductivity, differences among treatments were analyzed separately at each time point using one-way analysis of variance followed by Duncan’s multiple-range test. These time-point-specific analyses do not test an overall treatment-by-time interaction. Differences were considered significant at *p* < 0.05. Duncan’s test was used for within-analysis multiple comparisons; this does not imply control of an experiment-wide false discovery rate across endpoints or time points. Figures were prepared in Origin 2024. Concentration–response modelling and EC_50_ estimation were performed using the drc package in R, as described in Section 2.2.1. RNA-seq candidate-gene screening and functional over-representation analyses used unadjusted *p* values, as described in Section 2.2.10, and were interpreted separately as exploratory analyses.

## 3. Results

### 3.1. In Vitro Mycelial Growth Inhibition

**Figure 1:**
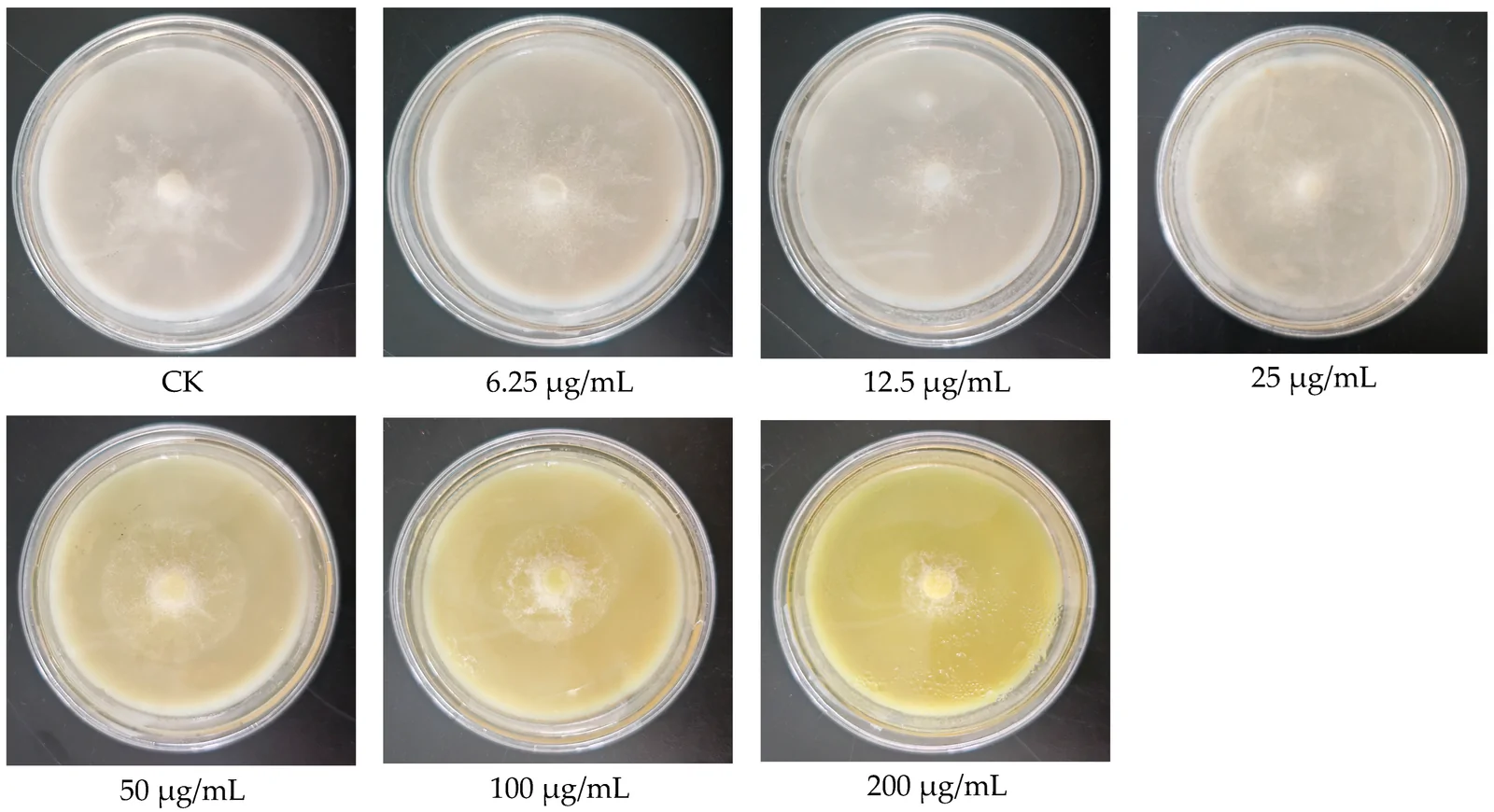
Concentration-dependent inhibition of *P. nicotianae* mycelial growth by berberine. Representative colonies were photographed after 4 d of incubation. Panels are arranged from left to right: CK, 6.25, 12.5, and 25 μg/mL in the upper row; 50, 100, and 200 μg/mL in the lower row. CK, untreated control.

Berberine inhibited the radial mycelial growth of *P. nicotianae* in a concentration-dependent manner (Figure 1). At 200 μg/mL, mycelial growth inhibition reached 79.37%. The four-parameter log-logistic model yielded an absolute EC_50_ of 42.10 μg/mL (SE = 8.01 μg/mL; 95% CI: 7.66–76.55 μg/mL), with a pseudo-R2 of 0.9872 (Table 2). Based on these results, 42 μg/mL, approximating the estimated EC_50_, and 200 μg/mL, the highest concentration tested, were selected for subsequent physiological, biochemical, and transcriptomic analyses, except for the dry-weight assay described in Section 3.2. The wide confidence interval indicates uncertainty in the EC_50_ estimate; 42 μg/mL was used as a nominal treatment concentration and should not be interpreted as a precisely established potency threshold.

### 3.2. Mycelial Dry Weight

**Figure 2:**
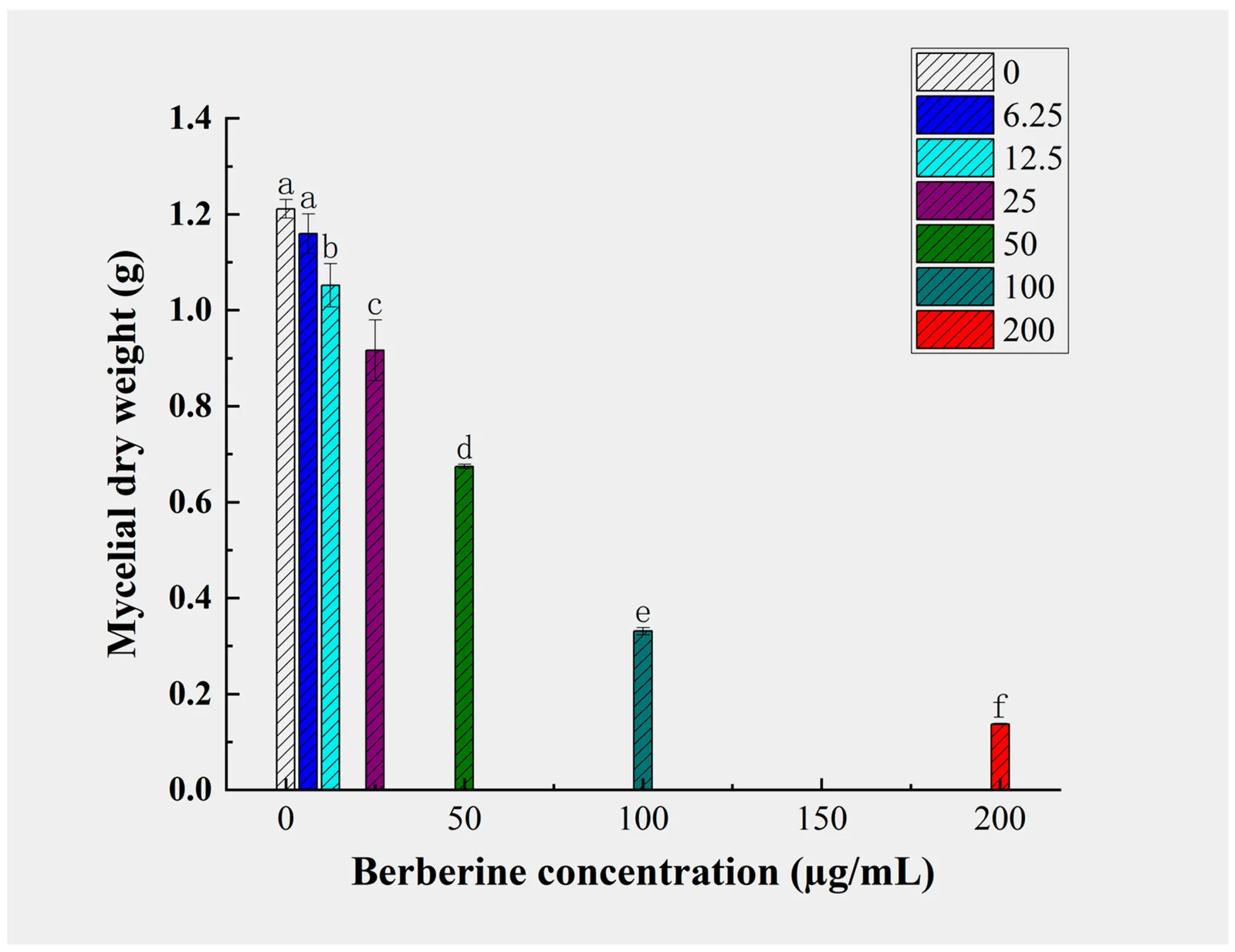
Effect of berberine concentration on the dry weight of *P. nicotianae* mycelia. Values are means ± SD (*n* = 3 independent biological replicates). Different lowercase letters indicate significant differences among treatments (one-way ANOVA followed by Duncan’s multiple-range test, *p* < 0.05).

After 7 d of exposure, berberine reduced the dry weight of *P. nicotianae* mycelia in a concentration-dependent manner (Figure 2). At 200 μg/mL, dry weight was 0.138 ± 0.002 g, compared with 1.211 ± 0.019 g in CK.

### 3.3. Malondialdehyde Content

**Figure 3:**
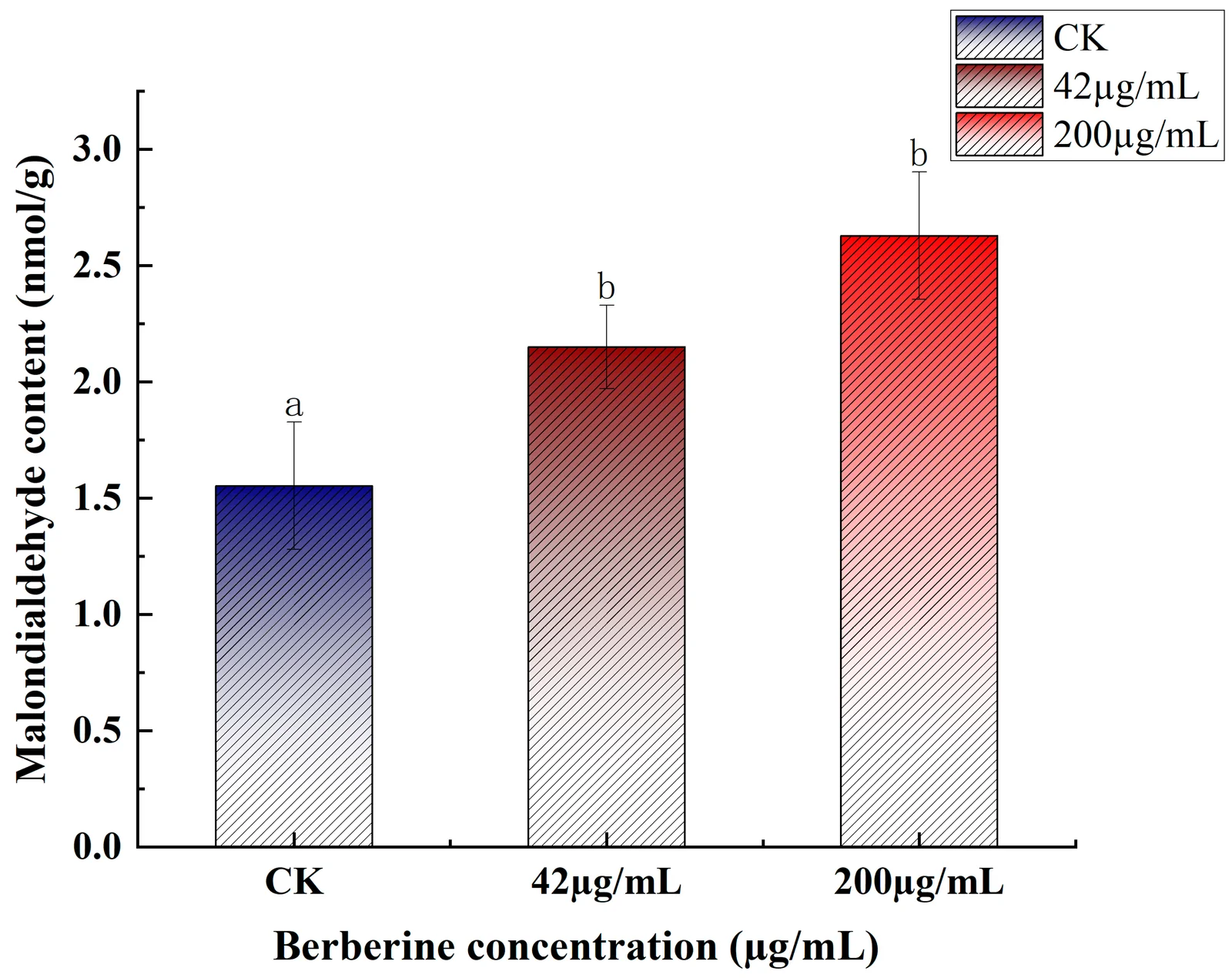
Effect of berberine on malondialdehyde (MDA) content in *P. nicotianae* mycelia. Values are means ± SD (*n* = 3 independent biological replicates). Different lowercase letters indicate significant differences among treatments (one-way ANOVA followed by Duncan’s multiple-range test, *p* < 0.05).

MDA content was significantly higher in both berberine-treated groups than in CK (Figure 3). At 200 μg/mL, MDA content reached 2.628 ± 0.273 nmol/g. Although the mean value was higher at 200 μg/mL than at 42 μg/mL, the difference between the two treatment concentrations was not statistically significant.

### 3.4. Antioxidant Enzyme Activities

After 24 h of berberine exposure, SOD activity in treated mycelia was significantly higher than that in CK, with the maximum value of 224.85 ± 4.93 U/g observed at 200 μg/mL. By contrast, mean POD activity was lower than CK and declined with increasing berberine concentration, reaching the minimum of 484.00 ± 24.25 U/g at 200 μg/mL. For CAT activity, the 42 μg/mL group showed a non-significant reduction relative to CK, whereas CAT activity at 200 μg/mL (122.40 ± 26.50 U/g) was significantly lower than both CK and the 42 μg/mL group (Table 3).

### 3.5. ATPase Activity and ATP Content

**Figure 4:**
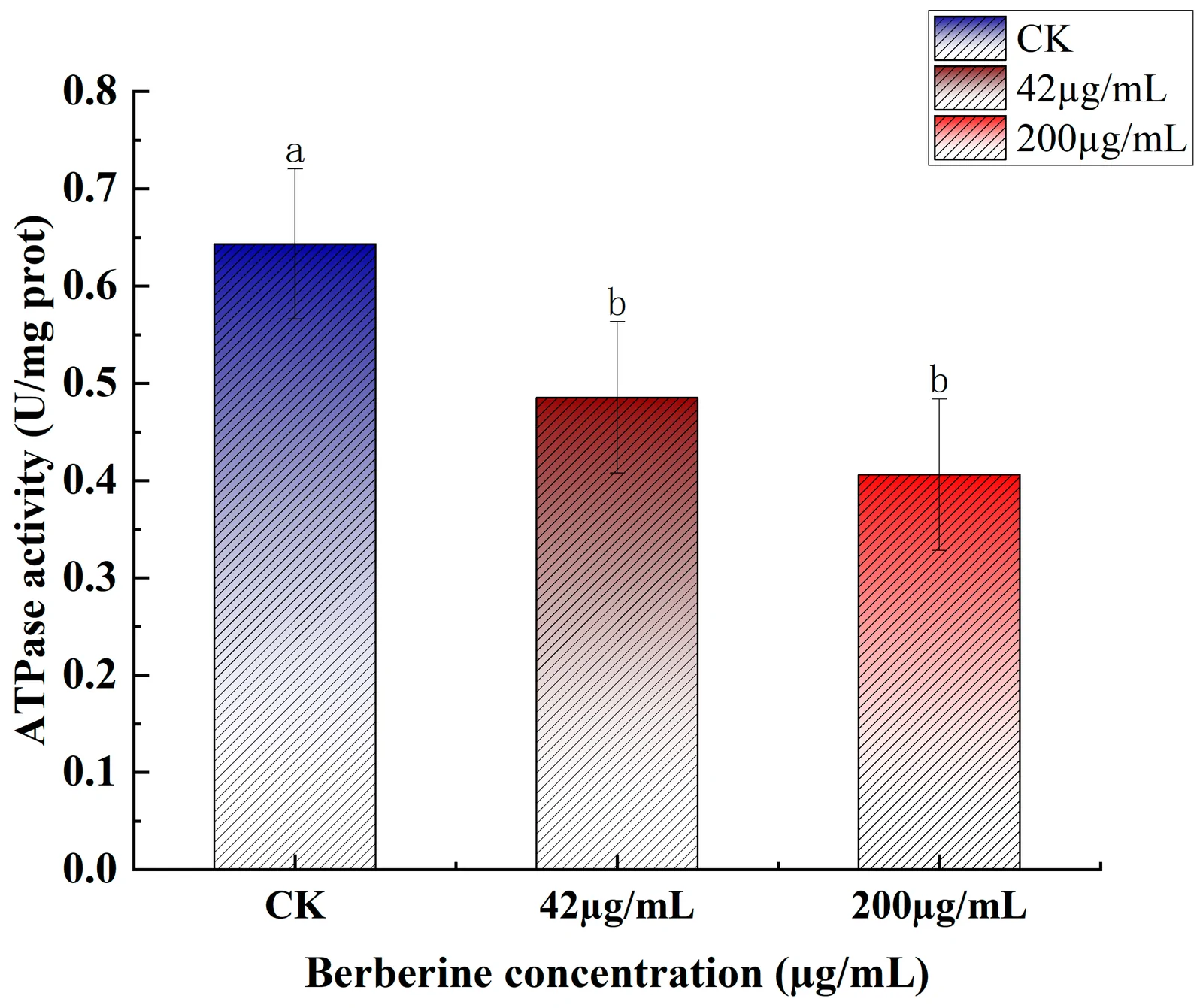
Effect of berberine on ATPase activity in *P. nicotianae* mycelia. Values are means ± SD (*n* = 3 independent biological replicates). Different lowercase letters indicate significant differences among treatments (one-way ANOVA followed by Duncan’s multiple-range test, *p* < 0.05).

**Figure 5:**
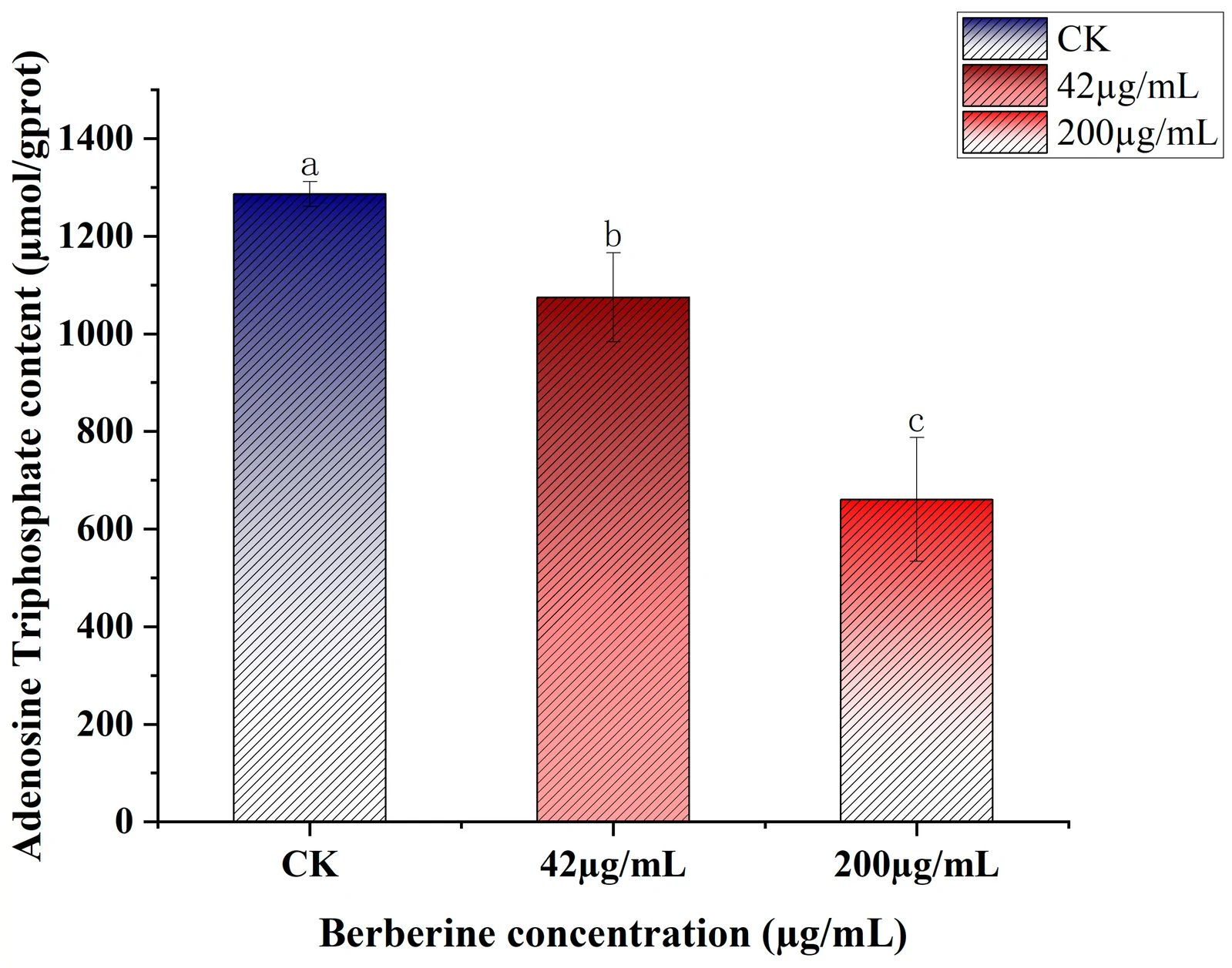
Effect of berberine on adenosine triphosphate (ATP) content in *P. nicotianae* mycelia. Values are means ± SD (*n* = 3 independent biological replicates). Different lowercase letters indicate significant differences among treatments (one-way ANOVA followed by Duncan’s multiple-range test, *p* < 0.05).

After 24 h of exposure, ATPase activity was significantly lower in both berberine-treated groups than in CK, with no significant difference between the two treatment concentrations (Figure 4). In contrast, ATP content decreased significantly from CK to 42 μg/mL and further to 200 μg/mL (Figure 5). At 200 μg/mL, ATPase activity and ATP content were 0.41 ± 0.08 U/mg protein and 660.6 ± 126.6 μmol/g protein, respectively.

### 3.6. Relative Electrical Conductivity

**Figure 6:**
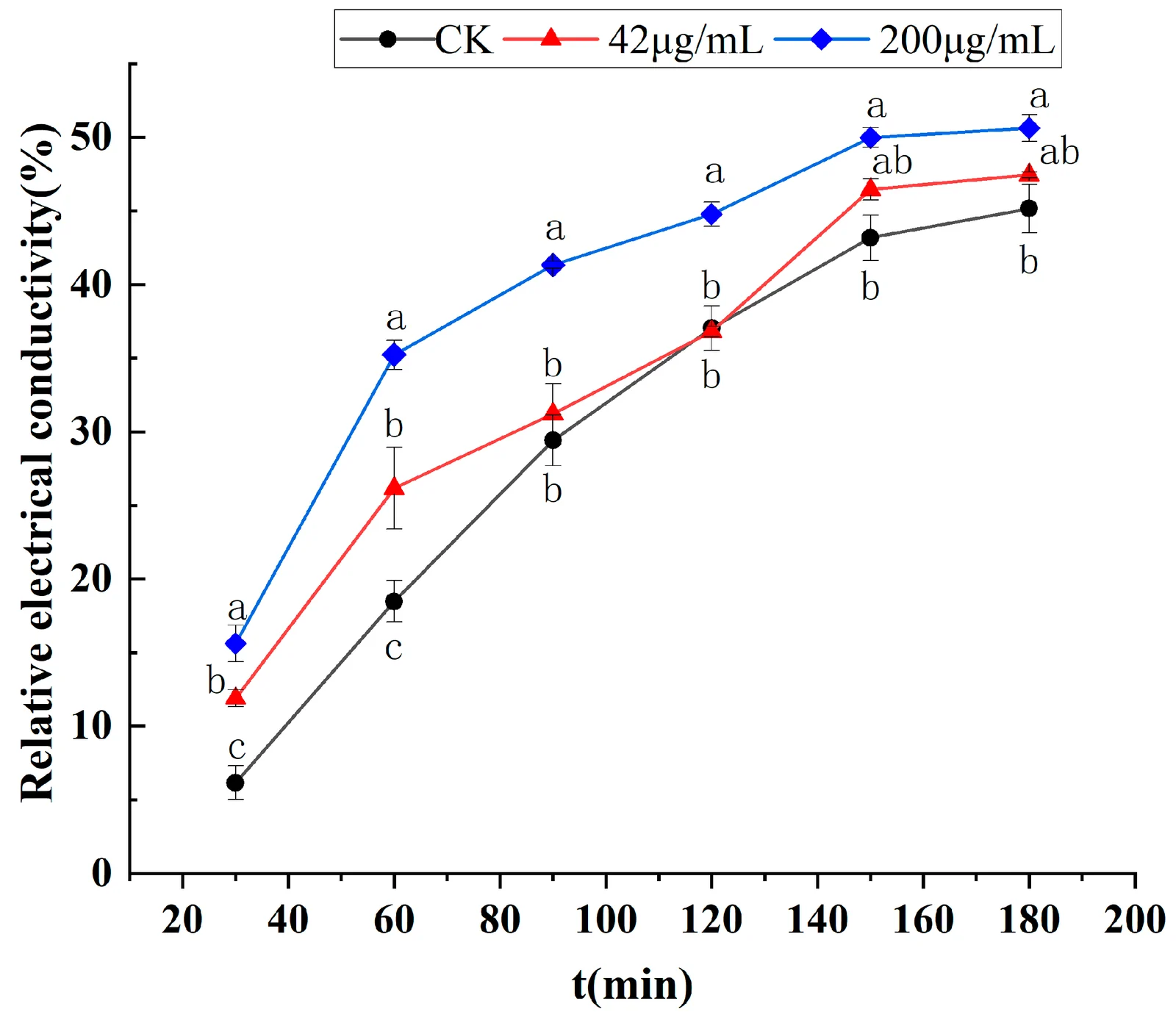
Changes in the relative electrical conductivity of *P. nicotianae* mycelia during exposure to berberine. Measurements were taken every 30 min for 180 min after immersion in distilled water containing 0 (CK), 42, or 200 μg/mL berberine. Values are means ± SD (*n* = 3 independent biological replicates). At each time point, different lowercase letters indicate significant differences among treatments (one-way ANOVA followed by Duncan’s multiple-range test, *p* < 0.05).

Relative electrical conductivity was measured at 30 min intervals for 180 min after mycelia were immersed in distilled water containing 0 (CK), 42, or 200 μg/mL berberine (Figure 6). Conductivity increased over time in all groups. Compared with CK, the 200 μg/mL group exhibited significantly higher relative electrical conductivity at every measured time point, whereas the 42 μg/mL group showed significantly higher values only at 30 and 60 min. At 180 min, relative electrical conductivity reached 47.45 ± 0.34% and 50.62 ± 1.55% in the 42 and 200 μg/mL groups, respectively. At this time point, the 200 μg/mL group differed significantly from CK, whereas the 42 μg/mL group did not differ significantly from either group.

### 3.7. Cell-Wall-Associated Staining

**Figure 7:**
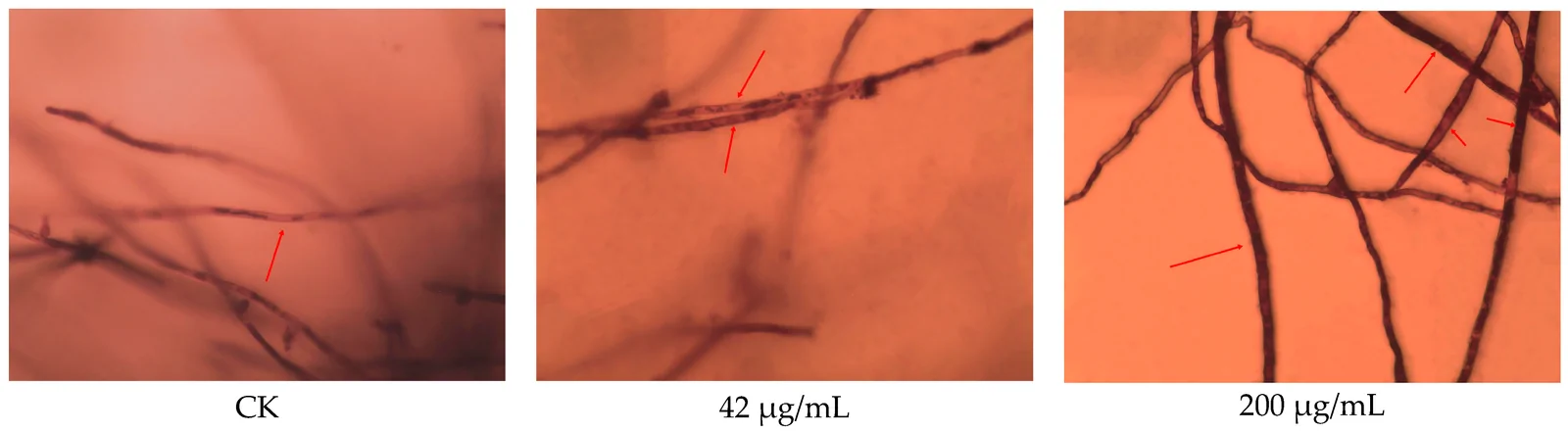
Sequential crystal violet and Congo red staining of *P. nicotianae* mycelia after 24 h of incubation without berberine (CK), 42, or 200 μg/mL berberine. Three independent biological replicates were used per treatment. Red arrows indicate stained hyphal regions. The images are presented for qualitative comparison only.

Sequential crystal violet and Congo red staining revealed clear differences between untreated and berberine-exposed mycelia (Figure 7). CK mycelia showed faint staining, whereas treated mycelia displayed progressively stronger intracellular purple staining as berberine concentration increased.

### 3.8. Propidium Iodide Staining

**Figure 8:**
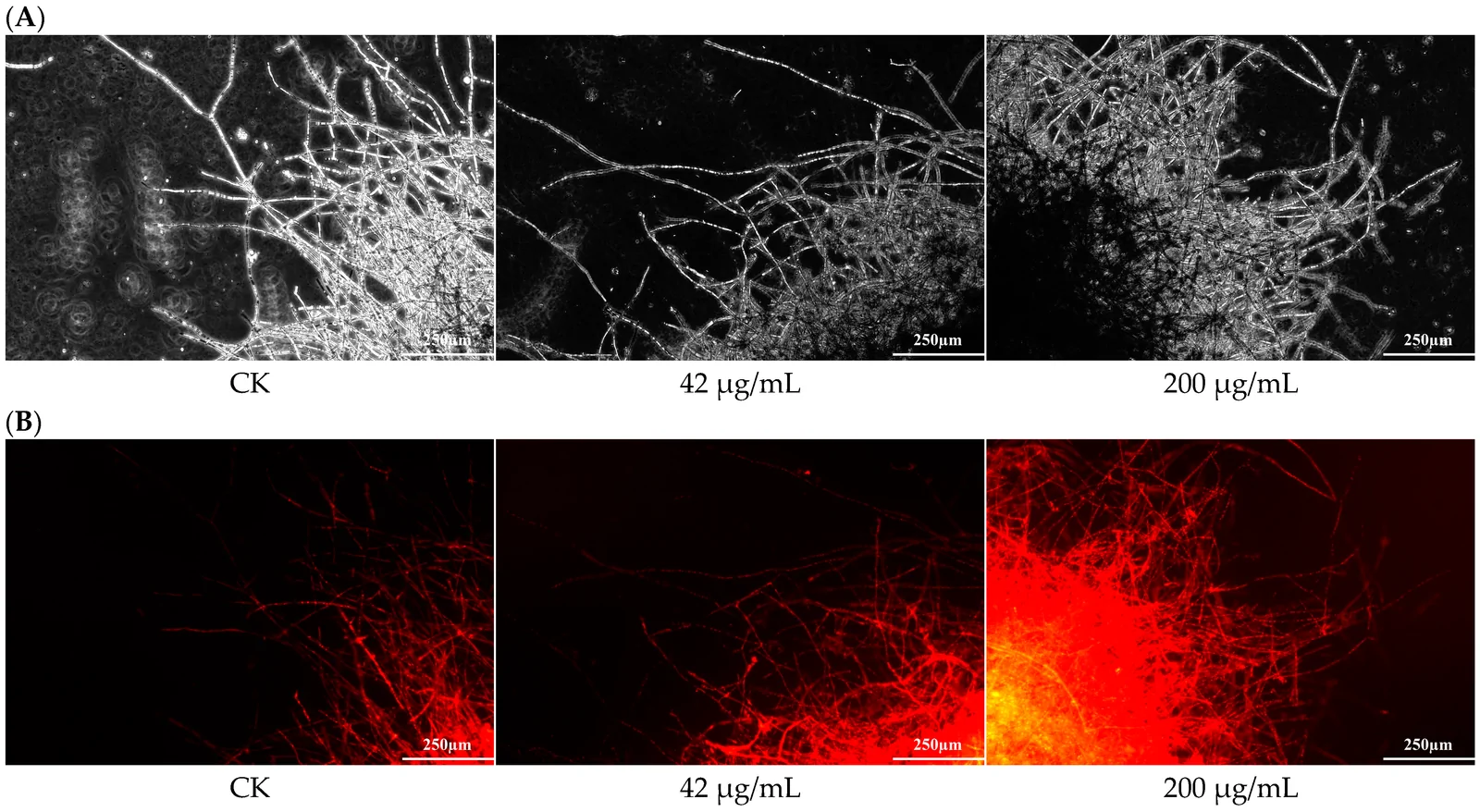
Propidium iodide (PI) staining of *P. nicotianae* mycelia incubated without berberine (CK) or with 42 or 200 μg/mL berberine. Columns show CK, 42 μg/mL, and 200 μg/mL, respectively. (**A**) Bright-field images, upper row; (**B**) PI fluorescence images, lower row. Within each treatment, the two images show the same field. Exposure and gain settings were consistent across treatments for each imaging mode. Three independent biological replicates were used per treatment. Scale bars = 250 μm. Representative images provide qualitative evidence of PI uptake; fluorescence intensity was not quantified.

PI-associated red fluorescence was weak in untreated mycelia and stronger in berberine-treated mycelia, particularly at 200 μg/mL (Figure 8). Paired bright-field and fluorescence images were acquired from the same field within each treatment under consistent acquisition settings. These qualitative observations are consistent with increased membrane permeability; fluorescence intensity was not quantified.

### 3.9. Scanning Electron Microscopy

**Figure 9:**
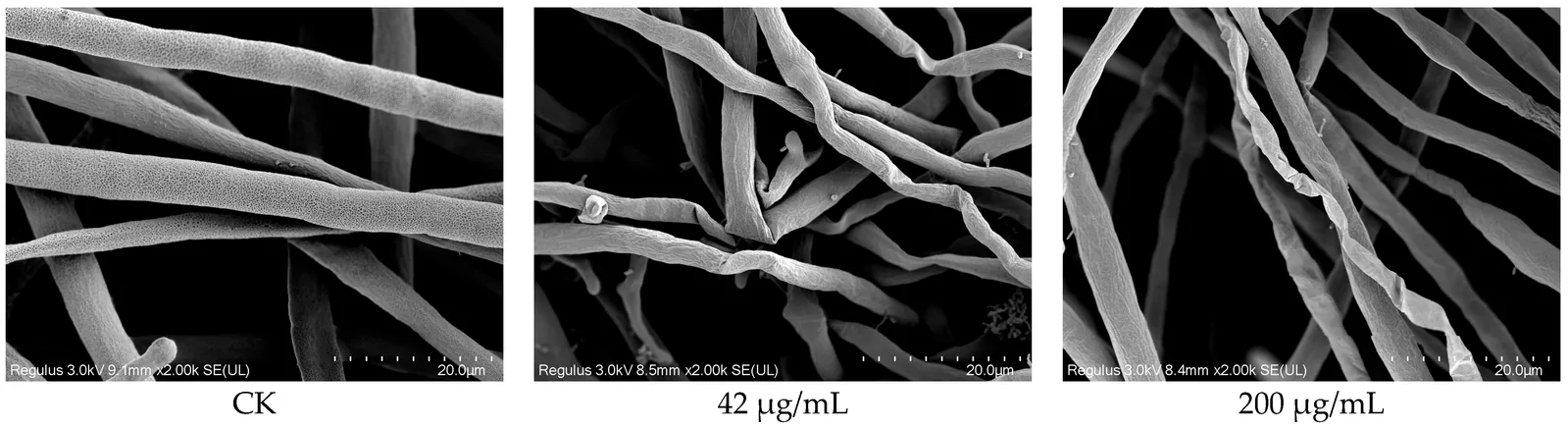
Scanning electron micrographs of *P. nicotianae* mycelia after 24 h of incubation without berberine (CK) or with 42 or 200 μg/mL berberine. Three independent biological replicates were used per treatment. Scale bars = 20 μm.

Scanning electron microscopy showed hyphae with smooth surfaces and regular contours in CK (Figure 9). Berberine caused shrinkage, surface folding, and collapse, and deformation was more severe at 200 μg/mL than at 42 μg/mL.

### 3.10. Transcriptome Sequencing

The nine RNA-seq libraries generated 364,800,730 raw reads from paired-end sequencing. After quality filtering, 361,085,704 clean reads were retained, corresponding to 98.91–99.03% of raw reads per library. Q30 proportions ranged from 95.43% to 95.80%, and GC content ranged from 55.84% to 56.46%. The total mapping rate was 94.90–95.61%; among mapped reads, 97.62–97.89% were uniquely mapped (Table S1).

**Figure 10:**
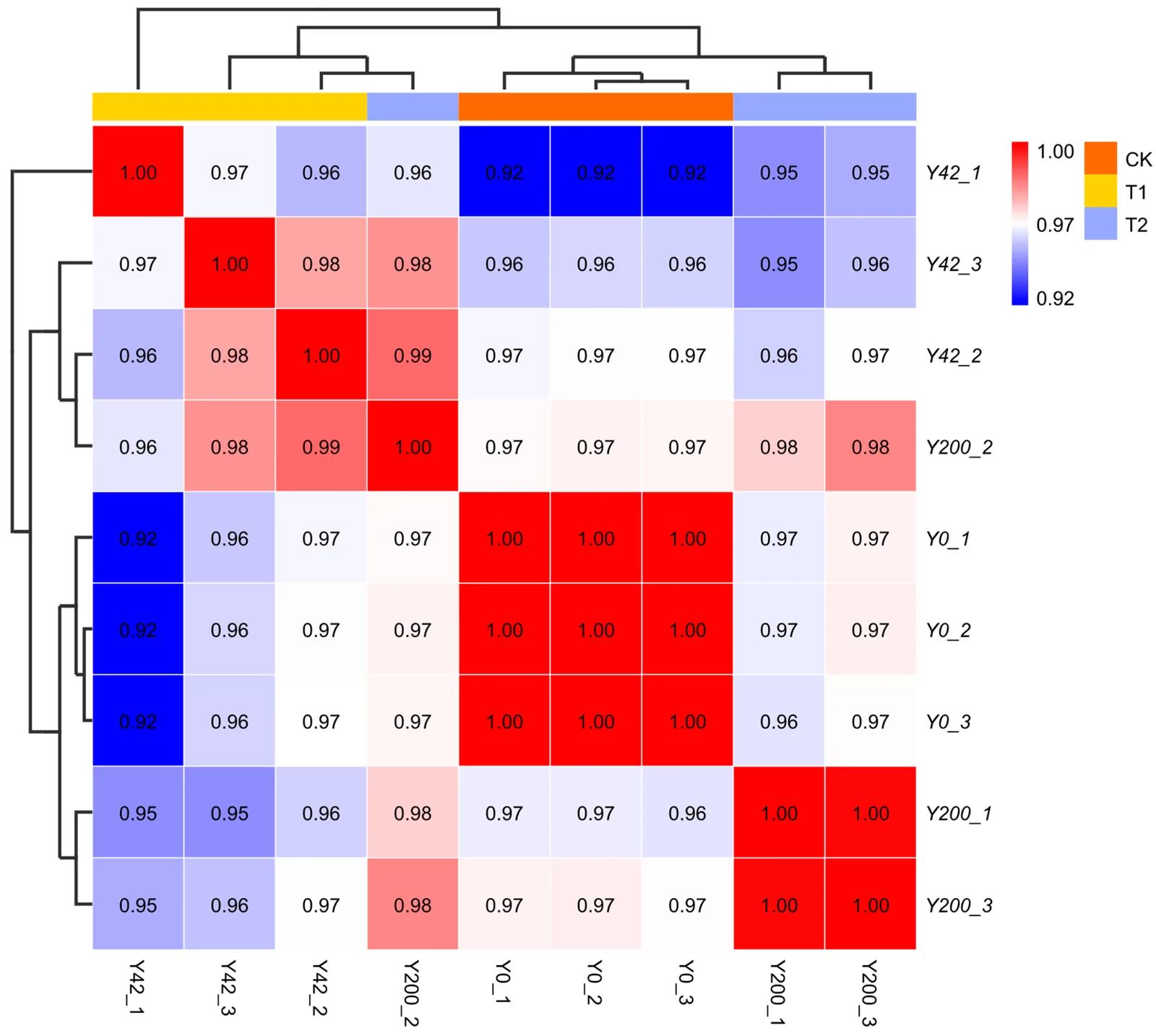
Pearson correlation heatmap of the nine RNA-seq libraries. Y0, untreated control; Y42, 42 μg/mL berberine; Y200, 200 μg/mL berberine. Three biological replicates were analyzed per treatment. Individual replicates are shown to assess sample-level consistency and variability rather than a treatment mean.

**Figure 11:**
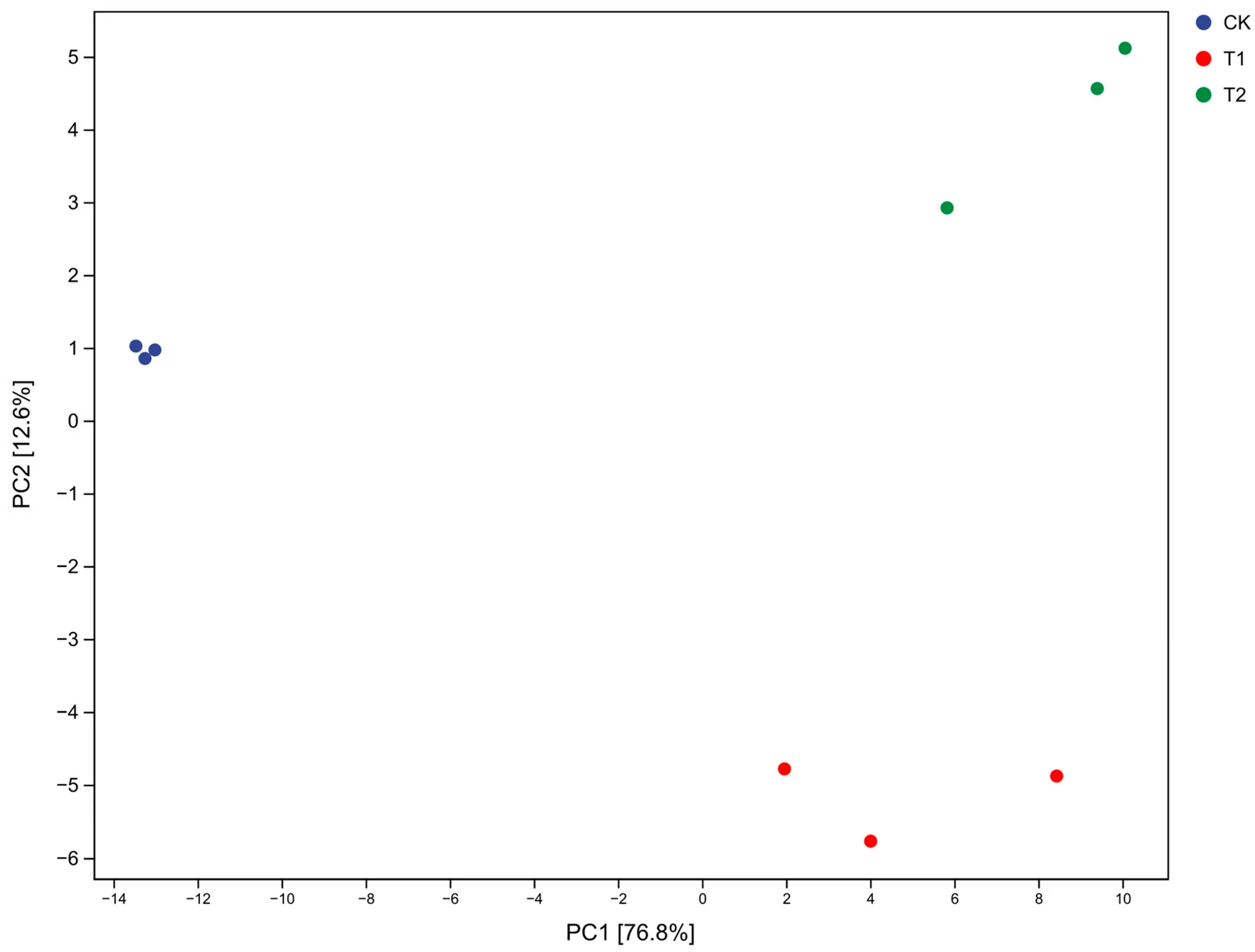
Principal-component analysis of the nine RNA-seq libraries. CK, untreated control; T1, 42 μg/mL berberine; T2, 200 μg/mL berberine. PC1 and PC2 explain 76.8% and 12.6% of the variance, respectively.

Pairwise Pearson correlation coefficients among the nine RNA-seq libraries ranged from 0.92 to 1.00 (Figure 10). The CK replicates clustered together, while Y200_2 clustered most closely with Y42_2. In the PCA plot, CK was separated from both berberine-treated groups along PC1, while the 42 and 200 μg/mL groups were separated along PC2 (Figure 11). The treated groups showed greater within-group dispersion than CK.

**Figure 12:**
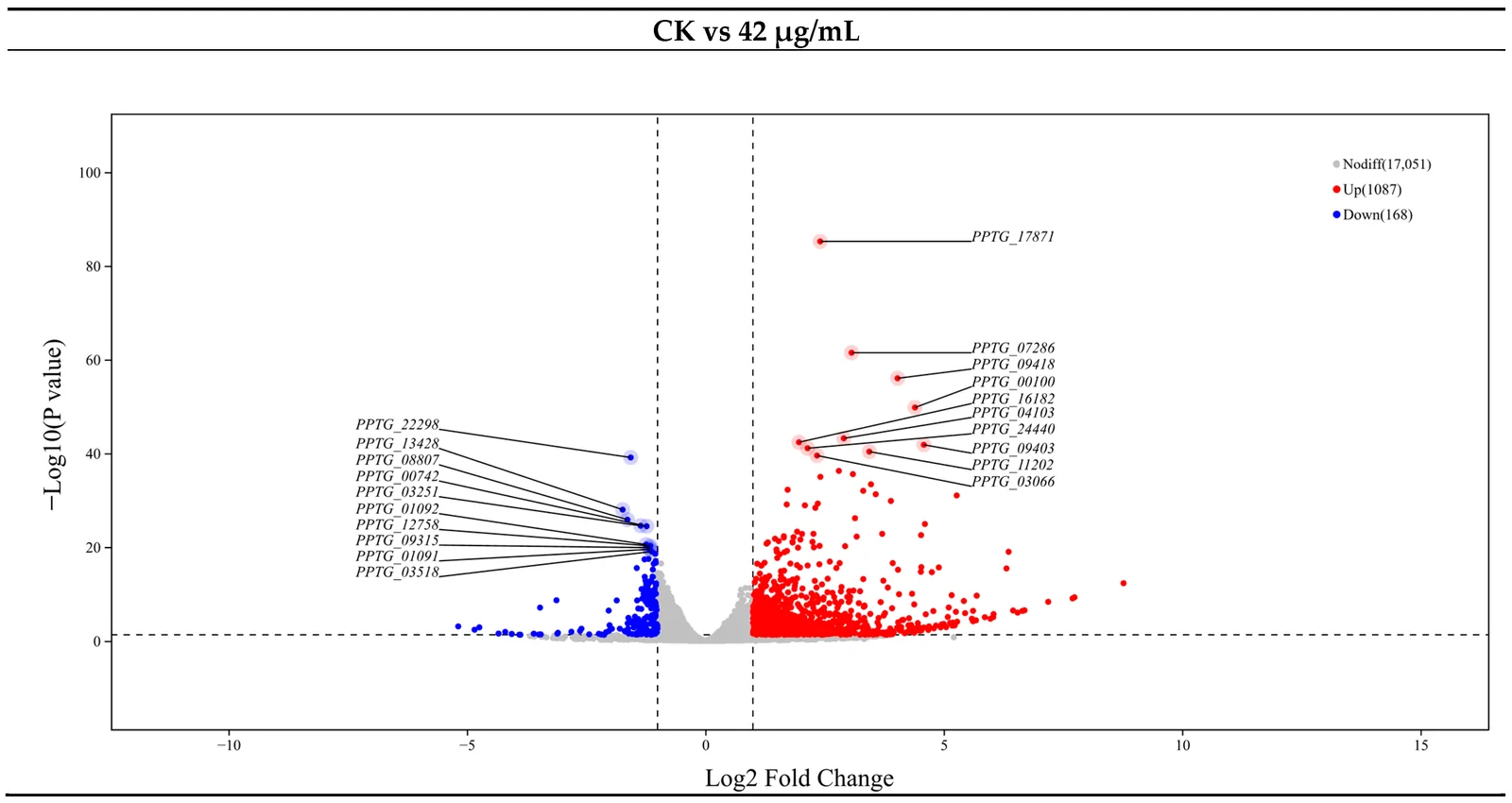
Exploratory differential-expression analysis of *P. nicotianae* exposed to 42 μg/mL berberine relative to CK. Each point represents a gene. The horizontal axis shows log2(fold change), and the vertical axis shows −log10(unadjusted *p* value). Candidate genes were screened using |log2(fold change)| > 1 and unadjusted *p* < 0.05. Red and blue points indicate upregulated and downregulated candidates, respectively; gray points indicate genes not meeting both criteria. Dashed vertical lines indicate |log2(fold change)| = 1, and the horizontal line indicates unadjusted *p* = 0.05. Three independent biological replicates were analyzed per treatment.

**Figure 13:**
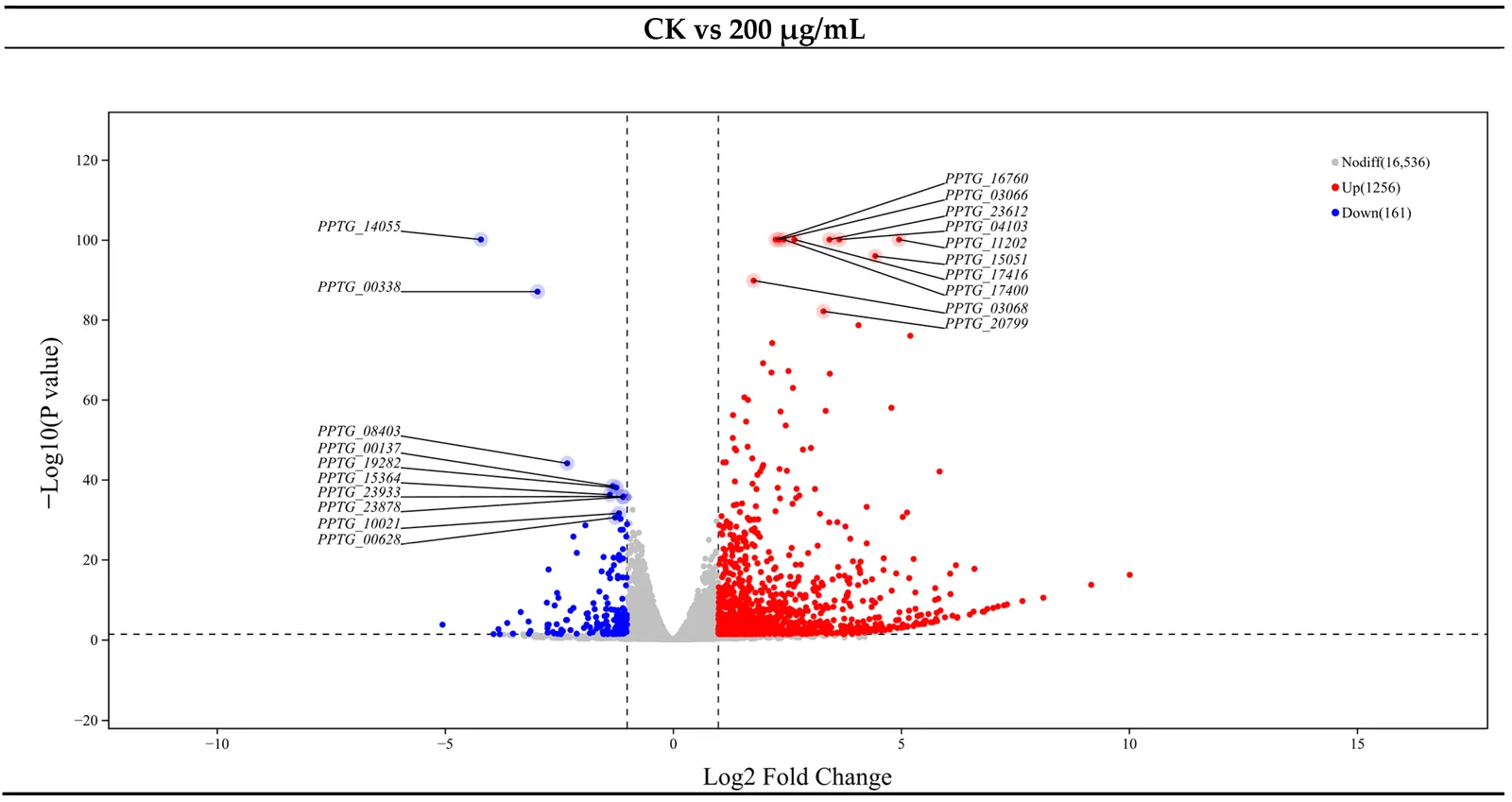
Exploratory differential-expression analysis of *P. nicotianae* exposed to 200 μg/mL berberine relative to CK. Each point represents a gene. The horizontal axis shows log2(fold change), and the vertical axis shows −log10(unadjusted *p* value). Candidate genes were screened using |log2(fold change)| > 1 and unadjusted *p* < 0.05. Red and blue points indicate upregulated and downregulated candidates, respectively; gray points indicate genes not meeting both criteria. Dashed vertical lines indicate |log2(fold change)| = 1, and the horizontal line indicates unadjusted *p* = 0.05. Three independent biological replicates were analyzed per treatment.

Exploratory screening using unadjusted *p* < 0.05 and |log2(fold change)| > 1 identified treatment-associated transcriptional changes in *P. nicotianae*. A total of 1255 candidate DEGs were identified at 42 μg/mL (1087 upregulated, 168 downregulated) (Figure 12), and 1417 candidate DEGs at 200 μg/mL (1256 upregulated, 161 downregulated) (Figure 13).

GO annotations were available for 508 and 364 candidate DEGs at 42 and 200 μg/mL, respectively. Exploratory over-representation analysis identified 20 nominally enriched terms for each concentration using unadjusted *p* < 0.05. At 42 μg/mL, prominent terms included microtubule-based movement, calcium ion binding, and potassium ion transport. At 200 μg/mL, prominent terms included calcium ion binding, polysaccharide metabolic process, lytic vacuole, and extracellular region. The detailed plots are provided in Figure S1. These findings were not supported by multiple-testing correction.

**Figure 14:**
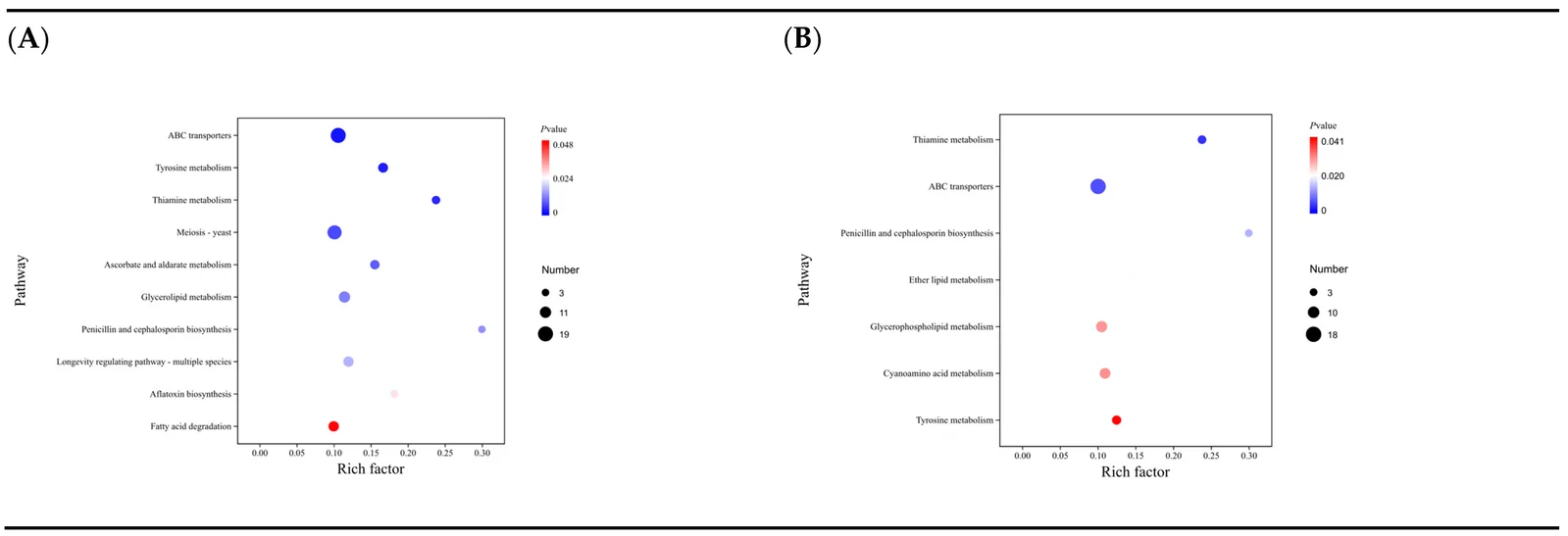
Exploratory KEGG over-representation analysis of candidate DEGs in *P. nicotianae* after berberine exposure: (**A**) 42 μg/mL versus CK; (**B**) 200 μg/mL versus CK. Candidate genes were screened using |log2(fold change)| > 1 and unadjusted *p* < 0.05. Pathway *p* values are nominal and unadjusted. Additional GO/KEGG plots and expression clustering are provided in Figures S1 and S2, respectively.

KEGG annotations were available for 92 and 57 candidate DEGs at 42 and 200 μg/mL, respectively. In exploratory over-representation analysis, the highest-ranked categories were ABC transporters and tyrosine metabolism at 42 μg/mL, and thiamine metabolism and ABC transporters at 200 μg/mL (Figure 14). These nominal associations are hypothesis-generating because the *p* values are not adjusted for multiple testing; they do not establish pathway activation.

Hierarchical clustering of KEGG-annotated candidate genes revealed heterogeneous expression patterns across nine clusters (Figure S2). Genes assigned to the same functional category, including ABC transporters, did not necessarily share the same expression pattern. These descriptive clusters summarize relative gene expression and should not be interpreted as direct measurements of pathway activity.

### 3.11. Targeted qRT-PCR Analysis

Eight genes, including *PPTG_00100* (CDC14A protein, XP_008889712.1), *PPTG_09403* (hypothetical protein, XP_008902651.1), *PPTG_09418* (ABCG36 protein, XP_008902665.1), *PPTG_15051* (TPK1 protein, XP_008910304.1), *PPTG_16182* (ACP6 protein, XP_008912046.1), *PPTG_16760* (hypothetical protein, XP_008912494.1), *PPTG_17400* (AP1M1 protein, XP_008913378.1) and *PPTG_21600* (Sod2 protein, XP_008896662.1), were selected for qRT-PCR for targeted comparison with the RNA-seq expression patterns.

**Figure 15:**
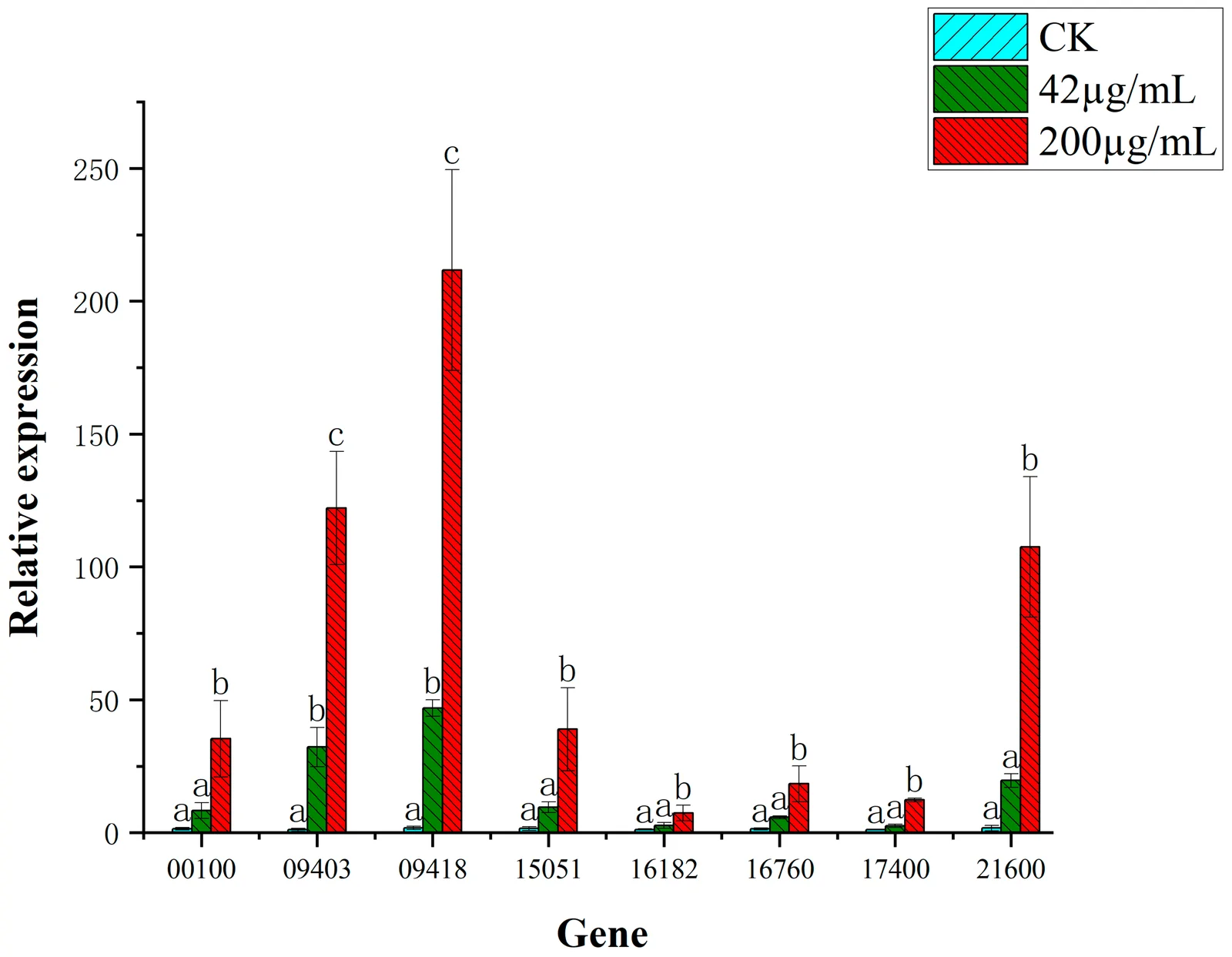
Targeted quantitative reverse-transcription PCR (qRT-PCR) analysis of eight candidate genes in *P. nicotianae* exposed to berberine. Expression was normalized to *PPTG_16002* (actin), with CK as the calibrator. Values are means ± SD (*n* = 3 independent biological replicates, each measured in three technical replicates). Within each gene, different lowercase letters indicate differences among treatments (one-way ANOVA followed by Duncan’s multiple-range test, *p* < 0.05).

qRT-PCR analysis showed that all eight selected genes had significantly higher relative expression at 200 μg/mL than in CK (Figure 15). At 42 μg/mL, significant increases relative to CK were observed for *PPTG_09403* and *PPTG_09418*, whereas the increases in the other six genes were not statistically significant. For all eight genes, relative expression was significantly higher at 200 μg/mL than at 42 μg/mL. The direction of the observed expression changes was consistent with the RNA-seq results, although the statistical significance of the qRT-PCR responses varied among genes and treatment concentrations. Because the same biological samples and a single reference gene were used, this agreement is a supportive cross-method comparison, not independent biological validation.

**Figure 16:**
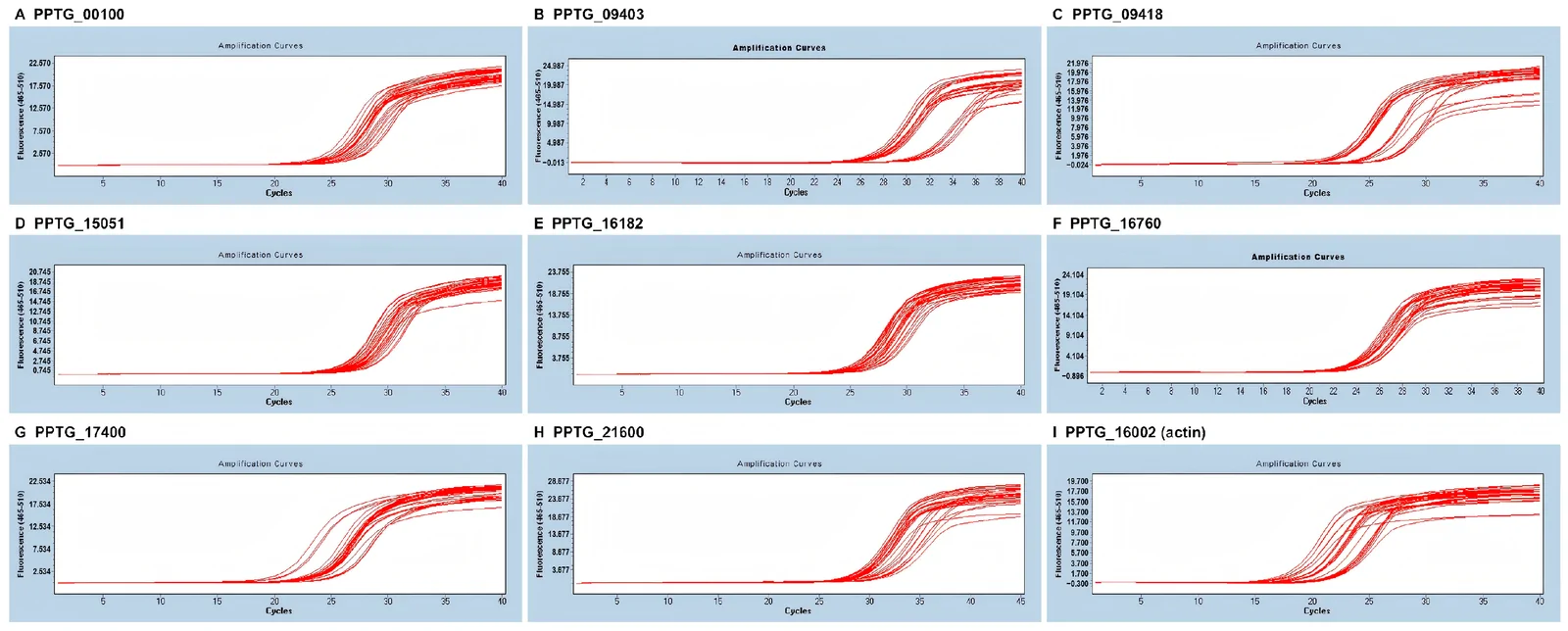
qRT-PCR amplification curves for the eight selected target genes and the reference gene.

**Figure 17:**
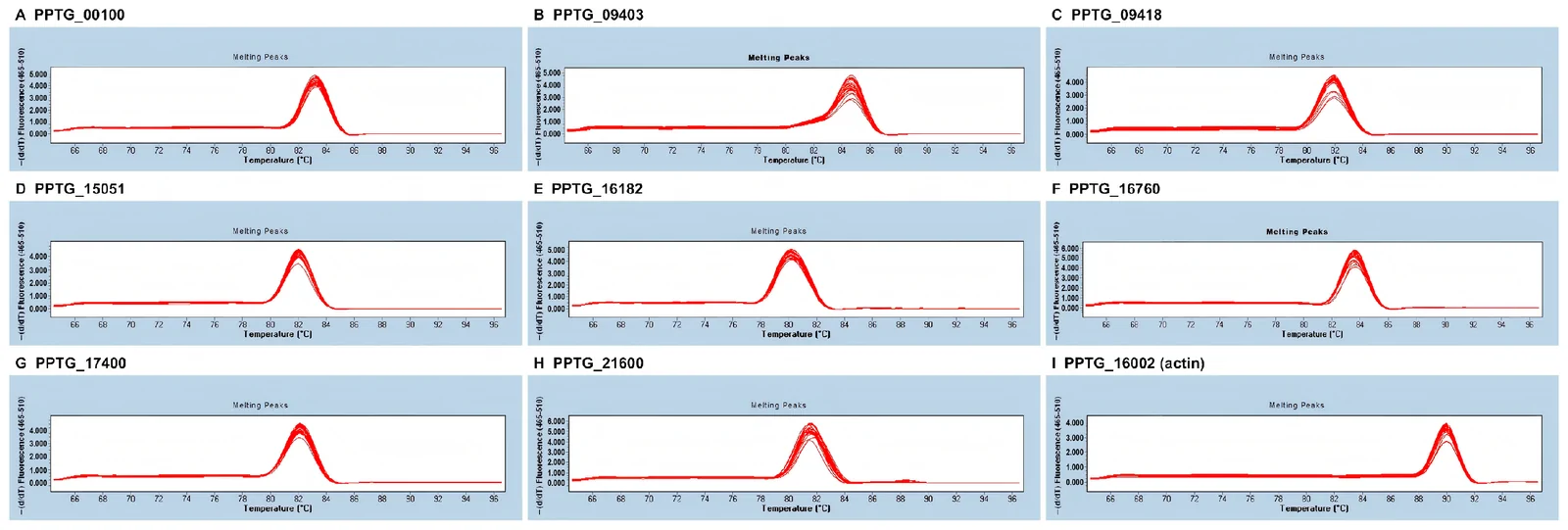
qRT-PCR melting curves for the eight selected target genes and the reference gene.

The amplification curves for the eight target genes and the actin reference gene showed consistent amplification behavior (Figure 16), while the corresponding melting curves supported product specificity (Figure 17).

## 4. Discussion

Berberine inhibited *P. nicotianae* growth in a concentration-dependent manner, with an estimated EC_50_ of 42.1 μg/mL (95% CI: 7.66–76.55 μg/mL). The model was fitted to mean responses at six nonzero concentrations; the wide interval limits the precision of potency comparisons and the interpretation of 42 μg/mL as an EC_50_-level exposure. Published studies provide context for this estimate, although direct potency comparisons are limited by differences in isolates and assay conditions. For example, honokiol exhibited an EC_50_ of 26.95 μg/mL against *P. nicotianae* [25], while chromone and apigenin 7-glucoside isolated from *Humulus scandens* exhibited EC_50_ values of 51.70 and 31.71 μg/mL, respectively [26]. These observations support further investigation of berberine as a candidate anti-oomycete compound; they do not establish comparative efficacy or disease control *in planta*.

The physiological and microscopy results support cellular-envelope damage following berberine exposure. Increased relative electrical conductivity and PI fluorescence were consistent with compromised membrane integrity. Crystal violet/Congo red staining revealed concentration-dependent changes in cell-wall-associated staining patterns, while scanning electron microscopy showed folded, shrunken, and collapsed hyphae, with more severe deformation at 200 μg/mL. Elevated MDA content provided additional evidence of lipid peroxidation-related changes accompanying these structural and permeability alterations. Together, these independent observations associate berberine exposure with cellular-envelope damage and hyphal deterioration. They do not establish whether lipid peroxidation-related changes precede membrane injury or arise as a consequence of it. The envelope alterations are broadly consistent with the effects reported for berberine and other botanical antimicrobial compounds against phytopathogens [9,15,19].

Berberine treatment was associated with differing responses among antioxidant enzymes in *P. nicotianae*. SOD activity increased significantly at both treatment concentrations, whereas mean POD activity was lower in treated groups. CAT activity was numerically lower at 42 μg/mL than in CK, but this difference was not statistically significant; at 200 μg/mL, CAT activity was significantly lower than in both CK and the 42 μg/mL group. These findings demonstrate enzyme-specific responses to berberine exposure. Together with elevated MDA content, they indicate changes in antioxidant enzyme activities and a lipid peroxidation-related marker, but do not directly establish the production or accumulation of reactive oxygen species. Whether the enzyme activity changes reflect direct effects of berberine or secondary physiological responses to cellular injury remains to be determined.

Berberine exposure was also associated with changes in the cellular energy status of *P. nicotianae*. ATPase activity and intracellular ATP content decreased, indicating altered ATP-related physiology and reduced ATP availability. Reduced ATP availability may constrain membrane maintenance, ion transport, and cellular repair, while cellular-envelope injury may increase the demands associated with stress adaptation. However, the present measurements do not establish the direction of these relationships or distinguish reduced ATP production from altered ATP consumption. The concurrent changes in ATP status, cellular-envelope integrity, MDA content, and antioxidant enzyme activities therefore support a multifaceted physiological response to berberine rather than a defined causal sequence. Energy-related responses have also been reported in *P. nicotianae* exposed to honokiol [25].

Exploratory transcriptomic screening identified candidate genes annotated to primary metabolism, lipid-related processes, transport, and stress responses. Genes assigned to alanine, aspartate and glutamate metabolism and starch and sucrose metabolism generally showed lower expression in treated samples, whereas genes assigned to glycerolipid metabolism; steroid biosynthesis; and cutin, suberine and wax biosynthesis showed higher expression (Figure S2). These gene-level patterns are compatible with responses accompanying reduced growth and altered cellular-envelope physiology, but they do not demonstrate changes in metabolic flux. Genes annotated to ABC transporters and ubiquinone and other terpenoid–quinone biosynthesis occurred in different expression clusters, indicating heterogeneous responses within these categories (Figure S2). The main nominal KEGG associations are summarized in Figure 14. Because candidate screening and over-representation analyses used unadjusted *p* values, these observations generate hypotheses rather than establish statistically confirmed enrichment, pathway activation, or causal mechanisms.

The assignment of three upregulated candidate DEGs at each concentration to the KEGG category “penicillin and cephalosporin biosynthesis” does not necessarily indicate activation of this biosynthetic pathway. KEGG pathway assignment alone does not demonstrate the presence of a complete biosynthetic pathway or the production of penicillin or cephalosporins in *P. nicotianae*. Further functional and metabolic analyses would be required to clarify the biological significance of this annotation. This annotation is retained in the supplementary KEGG plots for transparency (Figure S1).

The transcriptomic findings complement the physiological observations. Increased electrical conductivity and PI fluorescence supported membrane injury; cell-wall-associated staining and SEM revealed envelope-associated structural changes; elevated MDA content and altered SOD, POD, and CAT activities characterized lipid peroxidation-related changes and antioxidant enzyme responses; and reduced ATP content and ATPase activity indicated changes in ATP-related physiology. These observations occurred alongside expression changes in genes annotated to lipid metabolism, antioxidant and detoxification processes, transport, and primary metabolism. At the individual-gene level, the qRT-PCR expression estimates in genes annotated as CDC14A and AP1M1 identified candidates for investigating cell-cycle regulation and vesicular trafficking (Figure 15). Together, the data associate berberine exposure with cellular-envelope damage, altered energy status, and broad physiological stress responses. Nominal over-representation and homology-based gene annotations suggest potential functions, but do not demonstrate pathway activity or establish causal links among these responses. Direct cell-cycle, vesicle-trafficking, and gene-functional assays are required to test these hypotheses.

The scope of these findings is limited to one *P. nicotianae* isolate tested in vitro. Inter-isolate sensitivity, efficacy *in planta*, crop safety, formulation performance, and environmental fate remain untested. The EC_50_ estimate has a wide confidence interval. Candidate-gene screening and GO/KEGG over-representation analyses used unadjusted *p* values without false-discovery-rate control, so false-positive candidates and functional associations may be present. qRT-PCR used the same biological samples and a single reference gene and therefore provides supportive comparison rather than independent biological validation. Microscopy and physiological measurements establish associated changes but not their causal order. Multi-isolate studies, greenhouse and field evaluation, and targeted functional experiments are needed to assess biological mechanisms and practical disease-control potential.

## 5. Conclusions

Berberine inhibited the radial growth and reduced the biomass of the tested *P. nicotianae* isolate in vitro, with an estimated EC_50_ of 42.1 μg/mL (95% CI: 7.66–76.55 μg/mL). Exposure to 42 and 200 μg/mL berberine was associated with increased MDA content, SOD activity, and PI fluorescence, together with decreased POD and ATPase activities and ATP content. CAT activity decreased significantly only at 200 μg/mL. Relative electrical conductivity showed treatment- and time-dependent increases, and cell-wall-associated staining and SEM revealed changes in staining patterns and hyphal morphology. Exploratory transcriptomic analysis identified candidate differentially expressed genes annotated to primary metabolism, lipid and sulfur metabolism, ABC transporters, and antioxidant and detoxification processes using unadjusted *p* values. qRT-PCR on the same biological samples supported expression directions for eight selected genes but did not independently validate the exploratory findings. Collectively, these findings associate the in vitro inhibitory activity of berberine with antioxidant enzyme responses, lipid peroxidation-related changes, cellular-envelope damage, reduced ATP availability, and altered gene expression in *P. nicotianae*. The causal relationships among these responses and their relevance to disease control *in planta* remain to be established.

## Figures and Tables

**Table 1:** Primers used for targeted qRT-PCR comparison.

Primer Name	Sequence (5′–3′)
*PPTG_00100*-F	ATTCTCTTCCTTCCATCTT
*PPTG_00100*-R	CACCTTGAACTGATATGC
*PPTG_09403*-F	CAGTTCCTAGCACTGTGGCA
*PPTG_09403*-R	CGTTGAGGTATCGAGGGTCG
*PPTG_09418*-F	GTAAGAACGCCAGTAAGA
*PPTG_09418*-R	GGTTGTAGTTGTCCTCTA
*PPTG_15051*-F	TCTGTTATATGCTCGTGAA
*PPTG_15051*-R	GTTCTCCACTACCATCTC
*PPTG_16182*-F	GTCAACGCAGATAACAAT
*PPTG_16182*-R	GTCCTTCTTCATCTTCCT
*PPTG_16760*-F	AGTTGCAGACGGTCGACATT
*PPTG_16760*-R	GAGAAAGCCATCCAGCCAGT
*PPTG_17400*-F	CTTATGCTCATGTATCTCA
*PPTG_17400*-R	GAATACTCTCCTCTTCCA
*PPTG_21600*-F	AGCACATCAACACTGAAG
*PPTG_21600*-R	GCATCCTGAACCATCTTG
*PPTG_16002*actin-F4	CGTGACGAACTGGGATGACA
*PPTG_16002*actin-R4	GTACATGGCGGGTACGTTGA

**Table 2:** In vitro anti-oomycete activity of berberine against *P. nicotianae*.

Test Article	Concentration (μg/mL)	Colony Diameter (mm)	Inhibition Rate (%)	EC_50_ (μg/mL)	SE (μg/mL)	95%CI (μg/mL)	Pseudo-R^2^
Berberine	0	85.00 ± 0.0 a	0.00 g	42.1	8.01	7.66–76.55	0.9872
	6.25	84.58 ± 0.07 b	0.5 f				
	12.5	84.36 ± 0.14 c	0.76 e				
	25	65.39 ± 0.2 d	23.07 d				
	50	34.71 ± 0.06 e	59.16 c				
	100	29.38 ± 0.04 f	65.44 b				
	200	17.53 ± 0.05 g	79.37 a				

Note: Colony diameters are presented as means ± SD of three independent biological replicates. Different lowercase letters within a column indicate significant differences (*p* < 0.05). EC_50_ denotes the concentration causing 50% growth inhibition. SE, standard error of EC_50_; CI, confidence interval. Pseudo-R^2^ = 1 − RSS/TSS.

**Table 3:** Antioxidant enzyme activities in *P. nicotianae* after 24 h of berberine treatment.

Enzyme Activity (U/g)	CK	42 μg/mL	200 μg/mL
SOD	129.12 ± 5.29 a	163.85 ± 10.95 b	224.85 ± 4.93 c
POD	2740.00 ± 257.10 a	1930.00 ± 51.96 b	484.00 ± 24.25 c
CAT	321.30 ± 45.90 a	260.10 ± 26.50 a	122.40 ± 26.50 b

Values are means ± SD (*n* = 3 independent biological replicates). For SOD, POD and CAT, different superscript lowercase letters within a row indicate differences among treatments according to one-way ANOVA followed by Duncan’s multiple-range test (*p* < 0.05); letters are not comparable between enzymes. SOD, superoxide dismutase; POD, peroxidase; CAT, catalase; CK, untreated control.

## Data Availability

The raw RNA-sequencing data generated in this study are publicly available in the NCBI Sequence Read Archive (SRA) under BioProject accession number PRJNA1533711 (https://www.ncbi.nlm.nih.gov/bioproject/PRJNA1533711 (accessed on 23 September 2026)).

## Supplementary Materials

The following supporting information can be downloaded at: https://www.mdpi.com/article/10.3390/jof12100745/s1, Figure S1: Additional exploratory GO and KEGG over-representation plots; Figure S2: Expression patterns of KEGG-annotated candidate genes; Table S1: RNA-seq quality-control and mapping statistics for individual libraries.
