# Supplementary material for: In Vitro Inhibition of Phytophthora nicotianae by Berberine and Associated Physiological and Transcriptomic Responses
Source: J Fungi (Basel). 2026 Oct 4;12(10):745. doi: 10.3390/jof12100745 (PMC13706515; doi:10.3390/jof12100745)
Supplement: Supplementary file 1 [file jof-12-00745-s001.zip › jof-4616860-supplementary.pdf]

Supplementary Materials

In Vitro Inhibition of *Phytophthora nicotianae* by Berberine and Associated Physiological and Transcriptomic Responses

Table S1. RNA-seq quality-control and mapping statistics for individual libraries.

Sample	Raw reads	Clean reads	Q30 (%)	GC (%)	Total mapped (%)	Uniquely mapped (%)
Y0_1	44,182,444	43,737,150	95.60	56.46	95.61	97.73
Y0_2	37,617,538	37,207,592	95.43	56.46	95.55	97.73
Y0_3	39,873,552	39,456,150	95.43	56.44	95.57	97.73
Y42_1	40,990,208	40,571,954	95.77	56.00	95.42	97.71
Y42_2	42,264,656	41,829,244	95.80	56.08	95.08	97.75
Y42_3	42,895,342	42,455,254	95.55	56.18	94.90	97.62
Y200_1	40,042,138	39,654,854	95.62	55.84	95.49	97.89
Y200_2	38,589,412	38,207,614	95.68	56.00	95.06	97.81
Y200_3	38,345,440	37,965,892	95.73	55.93	95.49	97.88

Note: Uniquely mapped values represent percentages of mapped reads. CK, untreated control; Y42, 42 μg/mL berberine; Y200, 200 μg/mL berberine.

Figure S1. Additional exploratory GO and KEGG over-representation plots.

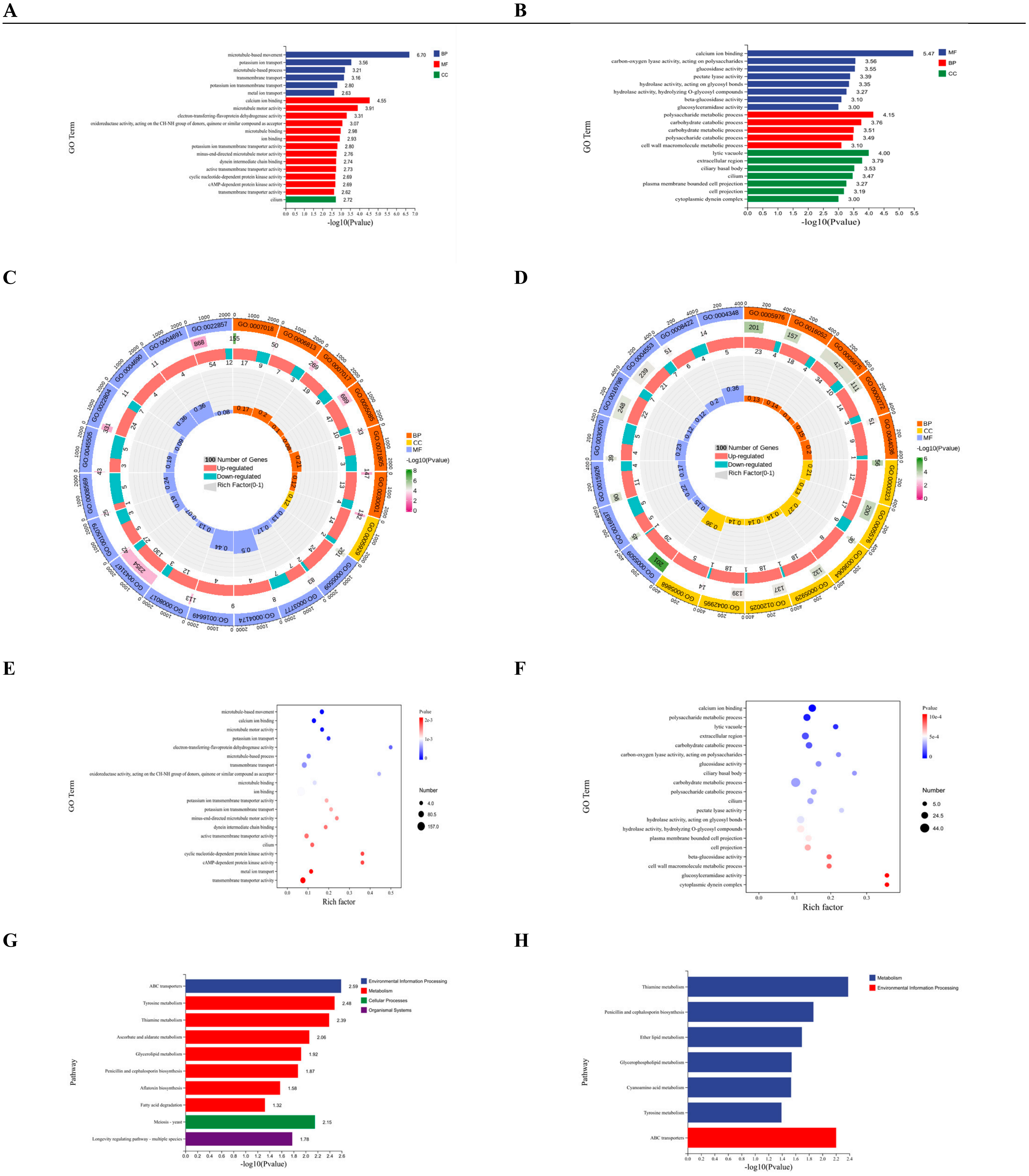

I

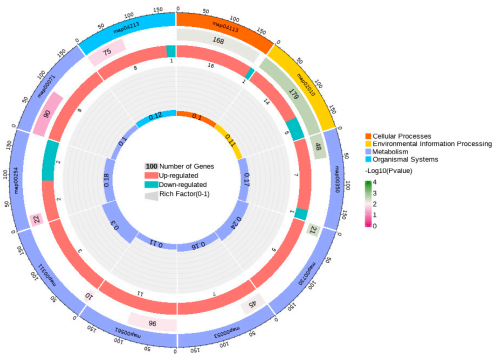

J

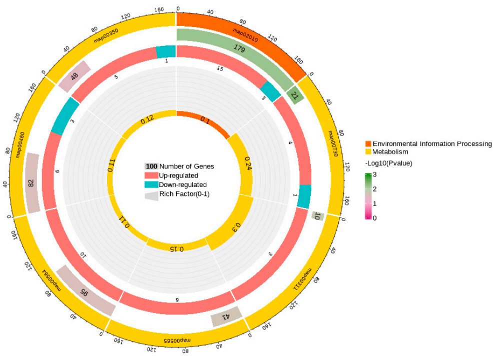

(A, B) GO bar plots; (C, D) GO circular plots; (E, F) GO bubble plots; (G, H) KEGG bar plots; (I, J) KEGG circular plots. In each pair, the first panel represents 42 μg/mL versus CK and the second 200 μg/mL versus CK. All enrichment P values are unadjusted and exploratory. Candidate screening used |log2(fold change)| > 1 and unadjusted P < 0.05. BP, biological process; MF, molecular function; CC, cellular component.

Figure S2. Expression patterns of KEGG-annotated candidate genes.

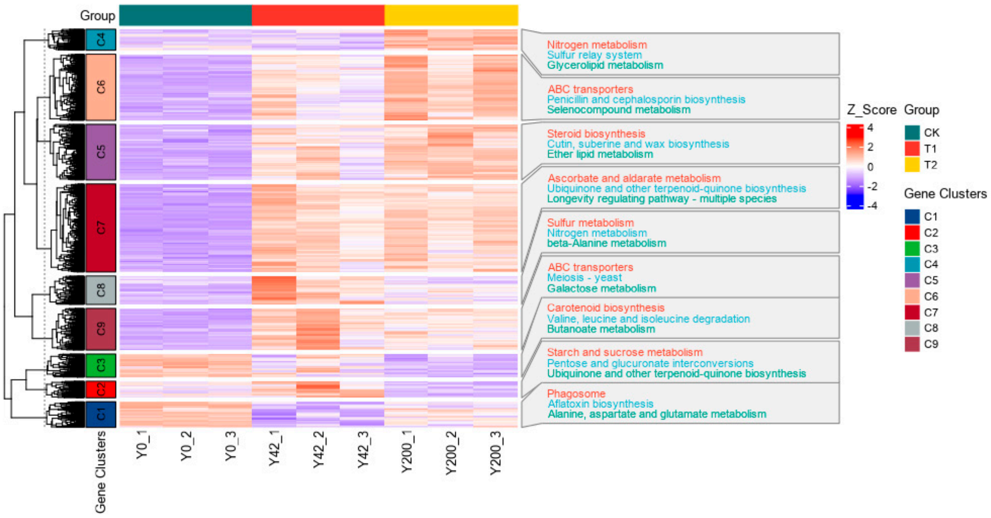

Heatmap of Z-score-transformed expression values with nine descriptive expression clusters (C1–C9). CK, untreated control; T1, 42 μg/mL; T2, 200 μg/mL. Candidate genes were selected using unadjusted P values. Relative expression patterns and KEGG annotations do not establish pathway activation.
